# The Roles of Left Versus Right Anterior Temporal Lobes in Semantic Memory: A Neuropsychological Comparison of Postsurgical Temporal Lobe Epilepsy Patients

**DOI:** 10.1093/cercor/bhx362

**Published:** 2018-01-17

**Authors:** Grace E Rice, Helen Caswell, Perry Moore, Paul Hoffman, Matthew A Lambon Ralph

**Affiliations:** 1 Neuroscience and Aphasia Research Unit (NARU), University of Manchester, Manchester, UK; 2 Department of Clinical Neuropsychology, Salford Royal Hospital, Manchester, UK; 3 Department of Clinical Neuropsychology, The Walton Centre NHS Foundation Trust, Liverpool, UK; 4 Centre for Cognitive Ageing and Cognitive Epidemiology (CCACE), Department of Psychology, University of Edinburgh, Edinburgh, UK

**Keywords:** anterior temporal lobectomy, conceptual knowledge, laterality, semantic memory, temporal lobe epilepsy

## Abstract

The presence and degree of specialization between the anterior temporal lobes (ATLs) is a key issue in debates about the neural architecture of semantic memory. Here, we comprehensively assessed multiple aspects of semantic cognition in a large group of postsurgical temporal lobe epilepsy (TLE) patients with left versus right anterior temporal lobectomy (*n* = 40). Both subgroups showed deficits in expressive and receptive verbal semantic tasks, word and object recognition, naming and recognition of famous faces and perception of faces and emotions. Graded differences in performance between the left and right groups were secondary to the overall mild semantic impairment; primarily, left resected TLE patients showed weaker performance on tasks that required naming or accessing semantic information from a written word. Right resected TLE patients were relatively more impaired at recognizing famous faces as familiar, although this effect was observed less consistently. These findings unify previous partial, inconsistent results and also align directly with fMRI and transcranial magnetic stimulation results in neurologically intact participants. Taken together, these data support a model in which the 2 ATLs act as a coupled bilateral system for the representation of semantic knowledge, and in which graded hemispheric specializations emerge as a consequence of differential connectivity to lateralized speech production and face perception regions.

## Introduction

Semantic memory (or conceptual knowledge) refers to our knowledge for the meanings of words, objects, people, and emotions (Lambon Ralph 2014; Lambon Ralph et al. 2017). There is now considerable, convergent evidence that semantic memory is supported by a large, distributed network of regions across the brain including the anterior temporal lobes (ATLs), bilaterally. The ATLs have been implicated as a transmodal representational “hub” for conceptual knowledge, with strong supportive data reported from patients with semantic dementia (SD) (Snowden et al. 1989; Mummery et al. 2000; Galton et al. 2001; Mion et al. 2010; Lambon Ralph et al. 2012), functional neuroimaging (Binney et al. 2010; Visser et al. 2010, 2012) and neurostimulation studies in healthy individuals (Pobric et al. 2007, 2010), and cortical-grid neurophysiological investigations (Shimotake et al. 2014). Whilst it is now established that semantic representation is supported through a bilateral ATL neural network, the nature and extent of the contribution from each ATL to semantic representation is unclear. Although the ATL atrophy in SD can be asymmetric in early cases, the disease is inherently bilateral and thus the individual contributions of left versus right ATL are hard to infer precisely from studies of this patient group. In contrast, however, en bloc resection for the treatment of temporal lobe epilepsy (TLE) provides an entirely unilateral model of left versus right ATL function. Accordingly, the current study comprehensively investigated a large-scale comparative case-series of patients with left versus right ATL resection.

Differences between left and right ATL function have been proposed based on: (1) the input modality, (2) the need for word retrieval, or (3) the social content of the stimulus (Gainotti 2007; Olson et al. 2007; Acres et al. 2009; Rice, Lambon Ralph, et al. 2015). The input modality account predicts differences between the left and right ATL functions based on the modality the stimulus is presented in. According to this view, the left ATL is relatively specialized for representing semantic information associated with verbal inputs (e.g., written and spoken words) and the right ATL for knowledge based on nonverbal, pictorial inputs (Snowden et al. 2004, 2012; Gainotti 2007; Olson et al. 2015). This is also consistent with the material-specific episodic memory hypothesis in TLE (Saling 2009; Willment and Golby 2013).

A second account suggests that the left ATL is relatively specialized for word retrieval tasks and the right ATL for visual recognition tasks (Tranel et al. 1997; Damasio et al. 2004). Evidence for this hypothesis was provided by Drane et al. (2012) who tested a group of presurgical and postsurgical TLE patients using a famous face naming task. Participants were asked to name a battery of famous faces and to provide specific semantic information as evidence of recognition. Drane et al. (2012) showed that both left and right TLE patients were impaired at naming famous people compared with a control group, but that left TLE patients were relatively more anomic compared with the right TLE group. In addition, patients with right TLE were impaired at correctly recognizing famous people. These findings are in line with previous studies suggesting a crucial role of the left ATL in lexical retrieval and for the right ATL in the visual recognition of famous people (Lambon Ralph et al. 2001; Seidenberg et al. 2002; Glosser et al. 2003; Drane et al. 2008, 2012; Mesulam et al. 2013).

Finally, a third account proposes differences between the left and right ATL based on social content, whereby the right ATL is relatively specialized for representing the meanings of social stimuli (Olson et al. 2007, 2013). This draws on previous evidence that the ATLs are involved in social cognition (Kluver and Bucy 1937; Edwards-Lee et al. 1997; Gallate et al. 2011) and explorations of whether part or all of the ATLs specifically code social concepts, including person knowledge and emotion concepts (Seidenberg et al. 2002; Glosser et al. 2003; Moll et al. 2005; Zahn et al. 2009; Skipper et al. 2011). In line with this hypothesis, in addition to their generalized semantic impairment, SD patients show deficits in social behavior including person recognition deficits, social awkwardness, and loss of empathy. These deficits can be much more apparent in the clinical presentation of SD patients with greater atrophy in the right hemisphere compared with the left (Thompson et al. 2003), though in a formal comparison, Chan et al. (2009) found that SD patients with greater left sided atrophy also exhibited social behavior deficits to a clinically significant degree. In the context of TLE, there have been inconsistent reports of deficits in both social behavior (Geschwind 1979; Bora and Meletti 2016) and emotion recognition after surgery (Sedda et al. 2013; Monti and Meletti 2015). Previous studies have also highlighted problems with person recognition after surgery; however, it is unclear whether this is related to the social relevance of person concepts or to visual recognition deficits (Seidenberg et al. 2002; Glosser et al. 2003; Drane et al. 2008, 2012).

To summarize, there are a number of ways in which the pattern of semantic deficits could differ in left and right postsurgical TLE patients. We tested these predictions in a large group of 40 TLE patients, using a comprehensive neuropsychological battery that probed verbal and visual semantic processing, word retrieval, person and face knowledge and emotion processing. Importantly, we measured both accuracy and reaction time wherever possible, greatly increasing the sensitivity of the test battery to subtle impairments and to fine-grained distinctions between left and right patients. A matched group of control participants was also used to assess whether there were deficits in both patient groups.

## Materials and Methods

### Patients

In total, 40 patients who had a single “en bloc” unilateral resection for TLE (20 left and 20 right TLE) were recruited from the neuropsychology departments at Salford Royal NHS Foundation Trust (Manchester, UK) and the Walton Center NHS Foundation Trust (Liverpool, UK) over a 14-month period. Eleven patients (6 left TLE, 5 right TLE) were reported in a previous study of postsurgical semantic memory performance (Lambon Ralph et al. 2012). All patients had long-standing epilepsy [age of diagnosis (years): left TLE; mean = 15.5, st. dev. = 7.4; right TLE; mean = 15.9, st. dev. = 7.9] stemming from unilateral hippocampal sclerosis. Patients with developmental disorders, head injuries, a history of psychiatric disorders, stroke or glioma or who had multiple epilepsy surgeries were excluded. All patients were right handed and native speakers of English. Table 1 summarizes the background demographic information. Both groups were matched in terms of age (*t*[38] = 0.76, *P* = 0.45), education level (*t*[38] = 0.25, *P* = 0.81), epilepsy duration (*t*[38] = 0.04, *P* = 0.96), time since surgery (*t*[38] = 1.48, *P* = 0.15) and the number of anti-epileptic medications (AEDs; *t*[38] = 0.68, *P* = 0.50). All patients were in the chronic phase postsurgery (left TLE; mean = 5.5 years, st. dev. = 4.5; right TLE; mean = 7.7 years, st. dev. = 4.1) and had a long-standing history of epilepsy (left TLE: mean onset = 21.8 years, st. dev. = 10.6; right TLE: mean onset = 21.1 years, st. dev. = 10.9).

**Table 1:** Demographic information for the control participants and the left and right TLE groups. Standard deviations are shown in parenthesis

	Age	Education (years)	Gender, M:F	Age at surgery (years)	Years since surgery	Age at diagnosis (years)	Epilepsy duration (years)	Volume resected (mm^3^)	# anti-epileptic medication (AEDs)
Controls	38.2 (12.2)	17.1 (2.2)	11:9	–	–	–	–	–	–
Left TLE	42.6 (11.0)	14.6 (2.5)	9:11	37.2 (10.8)	5.5 (4.5)	15.5 (7.4)	21.8 (10.6)	36.8 (12.0)	2.4 (1.2)
Right TLE	44.7 (10.1)	17.2 (3.0)	10:10	37.0 (10.1)	7.7 (4.1)	15.9 (7.9)	21.1 (10.9)	62.5 (19.7)	1.7 (1.3)

In all patients, histopathological analysis of the resection tissue revealed neuronal loss in the hippocampal region, consistent with a diagnosis of mesial temporal sclerosis. In line with the current neuropsychological literature, all patients reported impaired episodic memory, word-finding difficulties and significant lethargy at the end of the day. All patients were seizure free at the time of testing.

### Control Participants

Where available, performance of the left and right TLE patients was compared with published normative data. For all other tests, the performance of each TLE group was compared with 20 age-matched control participants (age: controls vs. left TLE: *t*[38] = 1.20, *P* = 0.24, controls vs. right TLE: *t*[38] = 1.96, *P* = 0.06; education: controls vs. left TLE: *t*[38] = 2.26, *P* = 0.03, controls vs. right TLE: *t*[38] = 1.98, *P* = 0.06). The TLE patients had completed marginally less formal education than controls, consistent with their long-standing neurological condition. All control participants were right handed, native English speakers. The experiment was approved by the local ethics board.

### Structural Scanning

In addition to the in-depth neuropsychological assessment, structural brain imaging data were acquired on a subset of 32 TLE patients (18 left, 17 right). Structural scans were acquired on a 3 T Phillips Achieva scanner, with a 32-channel head coil with a SENSE factor of 2.5. A high resolution T1 weighted structural scan was acquired for spatial normalization, including 260 slices covering the whole brain with TR = 8.4 ms, TE = 3.9 ms, flip angle = 8°, FOV = 240 × 191 mm^2^, resolution matrix = 256 × 206, voxel size = 0.9 × 1.7 × 0.9 mm^3^.

#### Automated Lesion Identification Procedure

**Figure 1.**
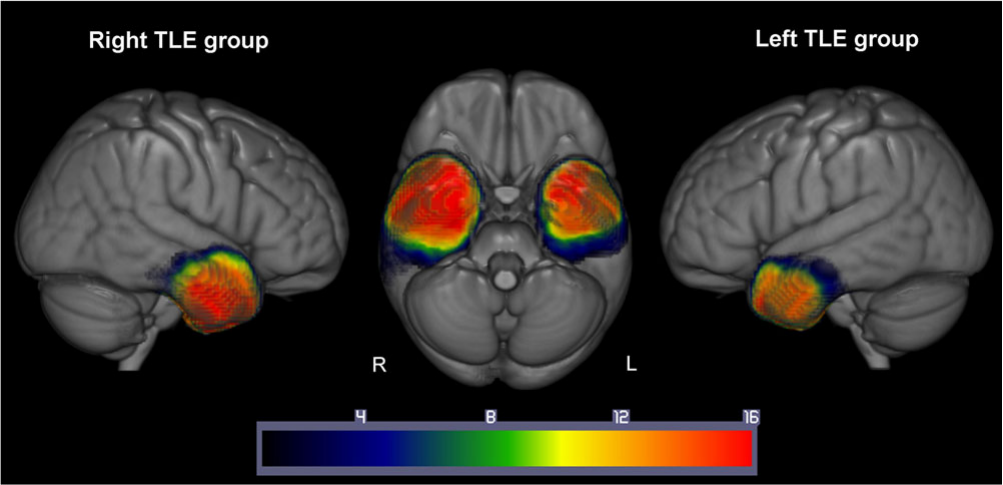
Resection overlap map for the 17 left and 17 right TLE patients. Overlap of the resection areas defined by the Seghier et al. (2008) method. Left TLE patients overlap is shown on the right of the image, right TLE patients overlap is shown on the left of the image. Color bars indicate the number of patients with resection in that area. Warmer colors = greater overlap, cooler colors = less overlap.

Automated outlines of the resection area were generated using Seghier et al.’s (2008) modified segmentation-normalization procedure, which is designed for use with brain-injured patients and which identifies areas of lesioned tissue. Data from both the TLE resected patients and the control participants were subjected to the automated lesion identification procedure. Segmented images were smoothed with an 8 mm full-width half maximum Gaussian kernel as recommended by Seghier et al. (2008) and submitted to the automated routines lesion identification and definition modules using the default parameters. The automated method involves initial segmentation and normalizing into tissue classes of grey matter, white matter, CSF and an extra tissue class which codes for the presence of the resection area. After smoothing, voxels that emerge as outliers relative to normal participants are identified and the union of these outliers provides the “fuzzy lesion map,” from which the resection outline is derived. The generated images were used to create the resection overlap map in Figure 1. For our sample, in order to ensure that the algorithm correctly identified the resection area as an extra class of tissue (rather than as CSF); the procedure was run twice for the TLE resected patients. The first iteration was run using the default settings in the toolbox; on the second iteration the default mask was changed to correspond to the output from the first iteration. This constrained the algorithm onto the resection area and allowed a more precise segmentation of the resection area.

### Neuropsychological Assessment

A detailed neuropsychological battery was designed to test, systematically, a broad range of semantic functions. Some tests were chosen because they have been used reliably to measure semantic performance in different patient groups, including TLE (e.g., synonym judgment, picture naming, word-picture matching), and in neurologically intact participants in neuroimaging and transcranial magnetic stimulation studies (Binney et al. 2010; Pobric et al. 2010; Visser and Lambon Ralph 2011; Visser et al. 2012). Additional semantic tests were included to assess the different accounts of left versus right ATL function. A number of tests were made more challenging (e.g., by including more foils/distractors or tapping into specific-level concepts rather than basic-level concepts) in order to be sensitive to the milder semantic problems presented in resected TLE patients (Lambon Ralph et al. 2012).

The majority of the neuropsychological tests were administered on a laptop running E-Prime software (version 1.2, Psychology Software Tools). Accuracy was measured for all tests and where possible reaction time data were also collected. Reaction times were calculated for all correct items within the test where possible; one left TLE patient’s reaction time data on the synonym judgment task had to be discarded because of a computer issue. Reaction time data for the Famous Face Naming task were not analyzed because these were too long and variable in control participants to be informative. All patients were able to complete the battery within one to 2 home visits, each lasting 2 h.

#### General Cognitive and Emotion–Social Function

To test general cognitive function the 2 subset forms of the Wechsler Abbreviated Scale of Intelligence battery (WASI: vocabulary and matrix reasoning), forward and backward digit spans, and the copy, immediate recall, and delayed recall of the Rey complex figure (Osterrieth 1944) were administered. To test for material-specific episodic memory impairments the face and word short form versions of the Camden Recognition Memory Battery (Warrington 1996) were administered. Two questionnaires to test aspects of social cognition were also utilized: (1) the Hospital Anxiety and Depression Scale (HADS) (Zigmond and Snaith 1983) to measure overall levels of anxiety and depression, and (2) the 81 item-Cambridge Behavioral Inventory-revised (CBI) (Wear et al. 2008) to measure behavioral change. This test was filled out by spouses/family members where possible. The CBI assesses 13 domains of behavior (e.g., memory, mood, motivation, and sleep). For each item, the spouse/family member must indicate how frequent the behavior is on a 5-point scale from never (0) to constantly (4). Out of the 13 scales, the 3 social subscales (e.g., stereotypical behavior, disinhibition, and abnormal behavior) and the mood subscale were of particular interest to the current study to explore possible lateralized effects in the right TLE group (Kumfor et al. 2016).

#### Semantic Functioning

To test semantic functioning, we included the 96-item synonym judgment test (Jefferies et al. 2009). This 3-alternative forced-choice (3AFC) task requires participants to match a probe word to one of 3 simultaneously presented alternative items. There is an orthogonal manipulation of word frequency (high, low) and imageability (high, medium, low) within this test. A difficulty-matched number judgment control task was also administered, which has also been used in TMS and functional neuroimaging studies (Pobric et al. 2007; Binney et al. 2010). Participants were presented with 3 numerical options and were required to pick the option which is numerically closest to the probe number. This test therefore provides a measure of nonsemantic processing speed.

A word-picture matching task was also administered, in which participants were required to state which picture matched the probe word (referring to a specific-level concept, e.g., “Dalmatian” and “robin”) from an array of 7 options (Rogers et al. 2015). The options were numbered and participants signaled their response by saying the corresponding number. Responses were recorded using a digital recorder. The onset of each trial was signaled using a beep, and correct response times were measured from the onset of the beep to the onset of the first proper response (i.e., ignoring filler responses).

#### Aspects of Semantic Knowledge Proposed to Show Hemispheric Lateralization

In addition to the semantic tests described above, a set of semantic tests designed to assess the functions of the left versus right ATLs was administered.

### Lexical and Object Decision

Lexical and object decision were included in order to test the predictions of the input modality hypothesis (Gainotti 2012). Previous research has proposed that both tasks require conceptual knowledge, particularly when the target cannot be distinguished from the foils on the basis of surface visual/orthographic features alone. Accordingly, SD patients show impaired performance on tests of lexical and object decision, particularly when the target is orthographically/visually atypical (e.g., animals which have distinctive features; Rogers et al. 2004; Patterson et al. 2006). Under these circumstances, surface associations cannot be relied upon and access to the correct semantic knowledge is needed.

We assessed lexical and object decision using more challenging, 4AFC versions of tests originally used by Rogers et al. (2004; stimuli kindly provided by Tim Rogers). For the 25-item lexical decision task, each 4AFC trial contained 1 real word and 3 nonwords (Supplementary Fig. S1, top). Each of the nonword alternatives had substantial orthographic similarity to the real word. For each trial, participants were required to pick out the real word from the 3 distractors. Each trial of the 25-item object decision task consisted of 4 line drawings, 1 depicting a real object/animal and 3 nonreal drawings of the same item (Supplementary Fig. S1, top). Participants were required to pick the real line drawing out from the 3 distractors.

### Specific-Level Picture Naming

Given that basic-level picture naming is not consistently impaired in resected TLE cases (Lambon Ralph et al. 2012), we chose to employ a more difficult picture naming task that would probe finer semantic distinctions. Specific-level concepts across a variety of categories (animals, flowers, cars, food, and clothing) were selected (Rogers et al. 2015). Verbal responses were measured using the same method as outlined for the word-picture matching test. Participants were instructed to give the most specific name they could for each item (e.g., “daffodil” rather than “flower”) and only subordinate-level responses were scored as correct.

### Famous Face Naming/Recognition

Previous studies have hypothesized a division of labor between the left and right ATLs for naming versus recognition of famous faces, respectively (Ellis et al. 1989; Seidenberg et al. 2002; Glosser et al. 2003; Drane et al. 2008, 2012). Thus, we included a 24-item famous face naming task following the protocol of Drane et al. (2012). Participants were asked to provide the first name and surname for each famous person where possible. If they could not produce the name, participants were encouraged to describe the individual and to be as specific as possible (e.g., why they were famous, occupation/nationality). Responses for this test were measured in the same way as the picture naming test described above. Scoring on this test was divided into a naming score and a recognition score, following the procedure outlined in Drane et al. (2012) (the only exception being that Drane et al. did not score trials in which participants reported being unfamiliar with the pictured person. We chose to include data for these trials on the basis that failure to report a person as familiar may be a consequence of impaired face processing.) As per Drane et al., any item that was correctly named was counted as successfully recognized as well as named. Any items where the name could not be produced but detailed semantic information could be recalled was counted as correct recognition only (e.g., responses such as “she’s a TV presenter” were considered too vague to constitute correct recognition for Cilla Black, whereas a response such as “she was the leading presenter of Blind Date, who died recently” was counted as sufficiently detailed to constitute correct recognition). Items where the name could not be recalled and only a vague sense of familiarity but no semantic information were counted as incorrect.

### Face-Name and Face-Description Matching

In addition to the expressive face naming task, a receptive 4AFC Face-Name/Face-Description matching task was also included (Supplementary Fig. S1, bottom). This was to determine whether any differences between left and right TLE were due to impaired name retrieval or to more general deficits in person knowledge. Different famous identities were included in the face naming and face-name matching tests in order to avoid priming effects. Each famous face was presented on screen and participants were required to make 2 decisions: (1) match the person to their correct name from 4 alternatives of the same gender and (2) match the face to the correct description again from 4 alternatives.

### Face Familiarity

To mirror the object and lexical decision tasks, a face familiarity test was presented using all the items from the famous face naming and the face-name matching tasks (81 items in total). Participants were required to pick out the famous face from 3 unfamiliar, but visually similar alternatives.

### Unfamiliar Face Perception

The short version of the Glasgow unfamiliar face test was administered (Burton et al. 2010) to test lower level face perception abilities. Participants were presented with pairs of unfamiliar faces in black and white, and asked to decide whether each face pair showed the same person or 2 different people. Previous studies have shown a relatively greater impairment in right TLE patients compared with left TLE patients on this test (Seidenberg et al. 2002; Glosser et al. 2003), which may be related to the famous face recognition problems previously reported.

### Emotion Recognition

The relative lateralization of emotion recognition is highly debated, including within the TLE literature (Monti and Meletti 2015). In a large-scale study of presurgical TLE patients Sedda et al. (2013) asked patients to categorize facial expressions from 5 different categories (happy, sad, anger, fear, and disgust), presented across 4 different actors (2 males and 2 females). In addition, there was also a manipulation of emotional intensity, ranging from the full expression (100% morph) to 35% of a neutral expression. The authors found that both left and right TLE patients exhibited deficits in categorizing all emotional categories compared with control participants, but that right TLE patients showed particular weaknesses in the negative emotion categories. Here, we replicated the task from Sedda et al. (2013): stimuli kindly provided by Anna Sedda, however, only the 100%, 75%, and 50% morphed images were included. The 35% morph images were excluded from the current study due to chance performance by control participants in previous studies (cf. Sedda et al. 2013). For brevity only the 100% morph results are reported in the main results below. Results from the other morphs are presented in the Supplementary Materials.

#### Camel and Cactus

In a follow-up study, we also had the opportunity to test the majority of the patients (17 left TLE; 17 right TLE) on a 2-alternative forced-choice version of the Camel and Cactus test (Bozeat et al. 2000). In this semantic task participants were presented with a probe item and 2 options, and were asked to pick the option which was most semantically related to the probe. Stimuli were presented as written words or pictures. This task and, in particular, the comparison of verbal versus nonverbal semantic performance provides an additional test of the input modality hypothesis (Gainotti 2012, 2014). Due to technical errors one left TLE patient had to be excluded from the accuracy analysis and 2 left TLE patients had to be excluded from the reaction time analysis.

## Results

### Structural Scans

Figure 1 shows the resection overlap maps for a subset of the left and right resected patients. Across both patient subgroups the area of resection was very consistent, with the anterior regions of the lateral and ventral ATL removed, along with the anterior portions of the hippocampus and amygdala. Overall, the right TLE patients resection volume was larger than that in the left TLE patients (left resected patients: 39.3 mm^3^ vs. right resected patients: 64.9 mm^3^; left > right: *t*[32] = 4.42, *P* < 0.0001), extending more posteriorly along the temporal lobes. This is in keeping with the current surgical standards whereby resections to the left hemisphere are more conservative to avoid disruption to the language centers (Wiebe et al. 2001).

### Results Summary

**Figure 2.**
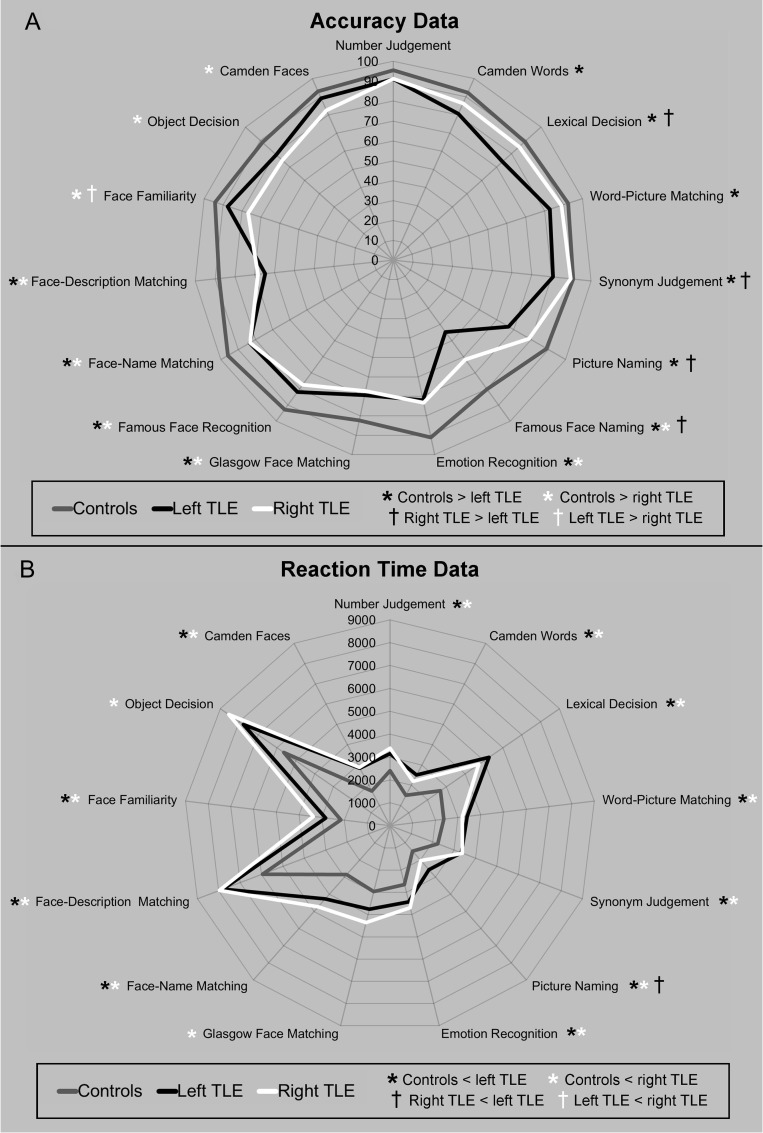
Summary of accuracy (A) and reaction time (B) data from the neuropsychological battery. Data for the control participants are shown in grey and data from the left and right TLE patients are shown in white and black, respectively. Accuracy data reported as percentages and correct response times are reported in milliseconds. Significant differences between the groups based on one-way between group ANOVAs are noted with asterisks; the color of the asterisk denotes the direction of the effect.

Performance across the 3 groups (controls, left TLE, right TLE) on the neuropsychological battery is displayed in Figure 2 in terms of accuracy (Fig. 2*A*) and response time (for correct items; Fig. 2*B*). Data from the WASI, the HADS and CBI are reported in Table 2. The analysis that follows focused on a main effect of group (controls, left, right TLE) using one-way between subjects ANOVAs. Unless otherwise stated, planned comparisons were run within each ANOVA to compare the control group against the left and right TLE groups separately, and to compare the left versus right TLE groups against each other (Table 2, Supplementary Table S1). Statistical significance was corrected for multiple comparisons using Bonferroni correction (*P* = 0.05/3 = *P* < 0.016).

**Table 2:** General cognitive function in TLE patients and control participants. Standard deviation shown in parenthesis. Comparisons between left and right TLE groups on the WASI and CBI were calculated with independent *t* tests. Comparisons between the control group and the TLE patients were calculated with a one-way between subjects ANOVA and individual effects were explored using planned comparisons. Planned comparisons are corrected for multiple comparisons using Bonferroni correction. Nonsignificant (n.s.) results are not reported for brevity

				Statistical comparison	
	Controls	Left TLE	Right TLE	Group main effect	Group comparisons
WASI:					
Overall	NA	91.9 (12.1)	101.4 (14.9)	NA	
Vocabulary		84.0 (18.2)	98.8 (8.4)		
Matrix reasoning		100.3 (13.7)	104.1 (11.4)		
Rey complex figure:					
Copy	35 (3)	35 (1)	35 (1)	n.s.	C > RTLE: *t*(57) = 2.87, *P* = 0.006
Immediate recall	21 (8)	15 (8)	14 (8)	*F*(2, 57) = 4.77, *P* = 0.012	C > LTLE: *t*(57) = 2.97, *P* = 0.004
Delayed recall	22 (9)	14 (8)	13 (8)	*F*(2, 57) = 6.85, *P* = 0.002	C > RTLE: *t*(57) = 3.40, *P* = 0.001
Digit span:					
Forward	6.7 (0.9)	6.2 (1.1)	6.8 (1.1)	n.s.	
Backward	4.5 (1.1)	4.5 (1.0)	4.2 (1.1)	n.s.	
HADS:					
Anxiety	5.7 (2.6)	7.0 (5.3)	7.1 (5.1)	n.s.	RTLE > C: *t*(57) = 3.47, *P* = 0.001
Depression	1.8 (1.7)	4.3 (3.6)	5.6 (4.5)	*F*(2, 57) = 6.23, *P* = 0.004	
CBI:					
Stereotypical behav.	NA	12 (7)	2 (3)	NA	n.s.
Disinhibition		2 (3)	6 (4)		n.s.
Abnormal behav.		3 (3)	3 (6)		n.s.
Mood		8 (7)	16 (11)		n.s.
Sleep		13 (4)	32 (12)		RTLE > LTLE: *t*(26) = 2.15, *P* = 0.04

An additional analysis also investigated the effect of resection volume on behavioral performance. As shown in Table 1, the resection volume for the right TLE group was on average 70% greater than the left TLE patients. Whilst in line with standard surgical protocols (Wiebe et al. 2001), this could cause a potential confound when exploring the results. Therefore, we ran additional analyses comparing behavioral performance in the left versus right TLE groups for whom we had T1 structural imaging (left TLE: *n* = 18; right TLE: *n* = 17). We first checked and confirmed that the pattern of behavioral results observed in the full patient sample held in this subset of patients. Then we ran ANCOVAs with resection volume as a covariate and again explored group difference between the left vs right resected patients. This was done for accuracy and reaction time data separately (see Supplementary Table S2).

### General Cognitive and Emotion–Social Function

Overall, the left TLE patients had lower IQ on the WASI, which was driven by lower IQ on the vocabulary subscale compared with the right TLE group. This may reflect anomia or deficits in verbal semantic processing. There were no differences between the left and right TLE patients on the matrix reasoning subscale. Patients performed within the normal range on the copy version of the Rey figure but the right TLE group was impaired at immediate recall and both groups were impaired at delayed recall compared with control participants. Patients also performed within the normal range on the digit span, both forward and backward (Table 2).

On the HADs, patients reported similar levels of anxiety compared with control participants; however, there was a tendency for the right TLE group to report greater levels of depression compared with controls (Table 2). This may be related to increased fatigue affecting some aspects of day to day life. In line with the existing literature and the Rey Figure recall scores, the most commonly reported behavioral change in both TLE groups on the CBI was memory problems. Of particular interest to the current study was the frequency of incidences on the social subscales (stereotypical behavior, disinhibition, and abnormal behavior) and the mood subscale. No differences in the frequency of behavior were shown in either TLE group on any subscale. The only difference between the left and right patient groups was more frequent sleep disturbances (Table 2).

### Episodic Memory

In line with the expectation from the current literature, patients demonstrated graded material-specific anterograde amnesia on the Camden Episodic memory tests (Warrington 1996). Left TLE patients were impaired in terms of accuracy on the word version of the test, while right TLE patients were impaired on the face version. Both groups were also significantly slower to respond than controls on both versions (Fig. 2, Supplementary Table S1).

### Semantic Memory in TLE

#### Synonym Judgment

**Figure 3.**
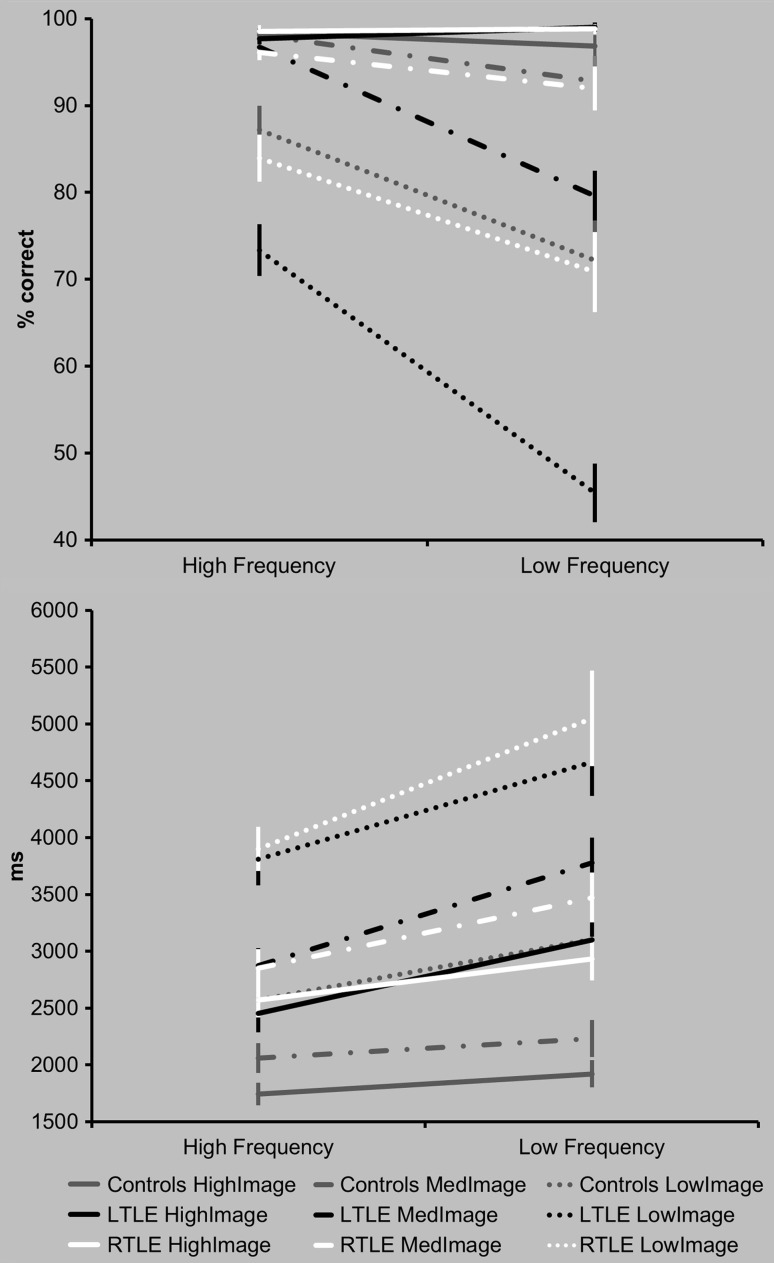
Synonym judgment frequency × imageability analysis. Breaking down performance on the synonym judgment task by frequency (high, low) and imageability (high, medium, low). The color of the line denotes the group (controls, grey; left TLE, white; right TLE, black). The line style denotes the condition (solid line = high imageability, hashed line = medium imageability, dotted line = low imageability). Error bars denote standard error.

Overall there were significantly slower responses in both patient groups compared with control participants. In terms of accuracy, there was a mild weakness in the left TLE group compared with the control group; no differences in accuracy were shown between the control group and the right TLE patients (Fig. 2, Supplementary Table S1). The synonym judgment task contains an orthogonal manipulation of word frequency (high, low) and imageability (high, medium, low). Figure 3 shows that in all 3 groups, the easiest condition (high frequency, high imageability) produced the highest accuracy and fastest reaction times. In terms of accuracy, both patient groups matched controls only on the easiest items. For the lower frequency, lower imageability items patients’ performance reduced, particularly in the left TLE patients. A similar pattern was shown for the reaction time data, although even on the easiest condition both patient subgroups were slower compared with controls. To investigate these patterns, the data were entered into a 3-way mixed ANOVA with main effects of Group (controls, left TLE, right TLE), frequency (high, low), and Imageability (high, medium, low). In terms of accuracy, the ANOVA confirmed a significant 3-way Group × Frequency × Imageability interaction (*F*[4, 114] = 4.32, *P* = 0.003). The ANOVA confirmed a significant main effect of Group (*F*[2, 57] = 9.18, *P* < 0.0001), as well as a significant Group × Frequency interaction (*F*[2, 57] = 6.67, *P* = 0.002). Further inspection of the high and low frequency items separately revealed that the interaction was driven by less accurate responses in the left TLE group compared with both the control participants and the right TLE group (for the most challenging items). For the high frequency items the left TLE patients were less accurate compared with control participants (*t*[57] = 3.12, *P* = 0.003). For the low frequency items the left TLE group were less accurate compared with control participants (*t*[57] = 3.74, *P* < 0.0001) and the right TLE group (*t*[57] = 4.26, *P* = 0.001). No differences in accuracy were found between the right TLE group and control participants for either high (*t*[57] = 1.41, *P* = 0.16), or low frequency items (*t*[57] = 0.26, *P* = 0.80). In terms of accuracy, the ANOVA also confirmed a significant Group × Imageability interaction (*F*[4, 114] = 9.74, *P* < 0.0001). Further inspection revealed the interaction was driven by the challenging medium and low imageability items—no group differences were shown for the (easier) high imageability items. For the medium imageability items, the left TLE group showed less accurate responses compared with both control participants (*t*[57] = 3.38, *P* = 0.001) and the right TLE group (*t*[57] = 2.76, *P* = 0.008). Similarly, on the low imageability items the left TLE group showed less accurate responses compared with the control group (*t*[57] = 4.16, *P* < 0.0001) and the right TLE group (*t*[56] = 3.73, *P* < 0.0001). No differences in accuracy were shown between the right TLE group and control participants in either imageability condition.

In terms of reaction times, a 3-way ANOVA confirmed a significant main effect of Group (*F*[2, 56] = 13.65, *P* < 0.0001). A significant Group × Frequency interaction was found (*F*[2, 57] = 5.73, *P* = 0.005). Further inspection revealed that for both high and low frequency items this effect was driven by slower reaction times in both patient subgroups compared with control participants (high frequency = left TLE vs. controls: *t*[56] = 4.17, *P* < 0.0001; right TLE vs. controls: *t*[56] = 4.62, *P* < 0.0001; low frequency = left TLE vs. controls: *t*[56] = 4.36, *P* < 0.0001; right TLE vs. controls: *t*[56] = 4.44, *P* < 0.0001). No significant differences between the 2 patient groups were found for either frequency condition. The ANOVA also confirmed a significant Group × Imageability interaction (*F*[4, 112] = 4.15, *P* = 0.004), further inspection also revealed slower reaction times across both patient groups compared with control participants (high imageability = left TLE vs. controls: *t*[56] = 4.12, *P* < 0.0001; right TLE vs. controls: *t*[56] = 4.16, *P* < 0.0001; medium imageability: left TLE vs. controls: *t*[56] = 4.77, *P* < 0.0001; right TLE vs. controls: *t*[56] = 4.28, *P* < 0.0001; low imageability: left TLE vs. controls: *t*[56] = 3.70, *P* < 0.0001; right TLE vs. controls: *t*[56] = 4.50, *P* < 0.0001). No significant differences between the patient groups were found for any imageability condition.

In contrast to the results of the synonym judgment test, there were no group differences in accuracy on the number judgment test. Both patient groups did, however, show significantly slower response times compared with control participants (Fig. 2, Supplementary Table S1). A 2-way ANOVA on reaction time data with main effects of Group (controls, left TLE, right TLE) and Task (synonym, number judgment) revealed no significant Group × Task interaction (*F*[2, 57] = 0.75, *P* = 0.48), indicating that the amount of slowing in the patients did not differ as a function of task. However, in terms of accuracy, there was a significant Group × Task interaction (*F*[2, 57] = 5.12, *P* = 0.009) driven by poorer responses in the left TLE group during the synonym judgment task. This indicates that the patients showed some slowing in reaction times but that the left TLE cases showed additional impairments in accuracy that were specific to the semantic task.

### Word-Picture Matching

Both patient groups were significantly slower to respond to specific-item word-picture matching trials than control participants. In addition, the left TLE group showed a weakness in terms of accuracy compared with controls (Fig. 2, Supplementary Table S1).

### Aspects of Semantic Knowledge Proposed to Show Hemispheric Lateralization

The following section describes the results from the semantic tests and tasks proposed to be semantically supported that have been previously hypothesized to engage the left and right ATLs differentially (Olson et al. 2007; Drane et al. 2012; Gainotti 2012; Sedda et al. 2013).

### Lexical and Object Decision

In keeping with the weakness in the left TLE patients on verbal tasks reported so far, left TLE patients showed decreased accuracy in lexical decision compared with controls. In the object decision task, the right TLE group showed decreased accuracy compared with control participants. For both tasks the right TLE group were significantly slower compared with control participants. In the left TLE group slower responses compared with control participants were observed in the lexical decision task only (Fig. 2, Supplementary Table S1). To test whether the left or right TLE groups showed relative impairments on the verbal/nonverbal tasks, a 2-way mixed ANOVA with a within-subject main effect of Task (lexical, object decision) and a between-subject main effect of Group (left, right TLE) was conducted. In terms of reaction time, no significant Task × Group interaction (*F*[1, 38] = 2.05, *P* = 0.16) was shown, suggesting that both groups were slowed equivalently across both tasks. In terms of accuracy, a significant Task × Group interaction (*F*[1, 38] = 10.22, *P* = 0.003) was driven by poorer performance in the left TLE group on the lexical decision task compared with the right (*t*[38] = 3.04, *P* = 0.005). The 2 groups did not differ on the object decision task, however, (*t*[38] = 1.06, *P* = 0.29). Therefore, these results provide partial support for the input modality hypothesis suggesting there is a relative weakness for processing semantic information presented as a verbal input (i.e., written word) after left ATL resection; however, there was no evidence for the corresponding effect for nonverbal semantic processing after right ATL resection. This is in line with the findings from a recent meta-analysis of fMRI data of neurologically intact participants (Rice, Lambon Ralph, et al. 2015).

### Specific-Item Picture Naming

**Figure 4.**
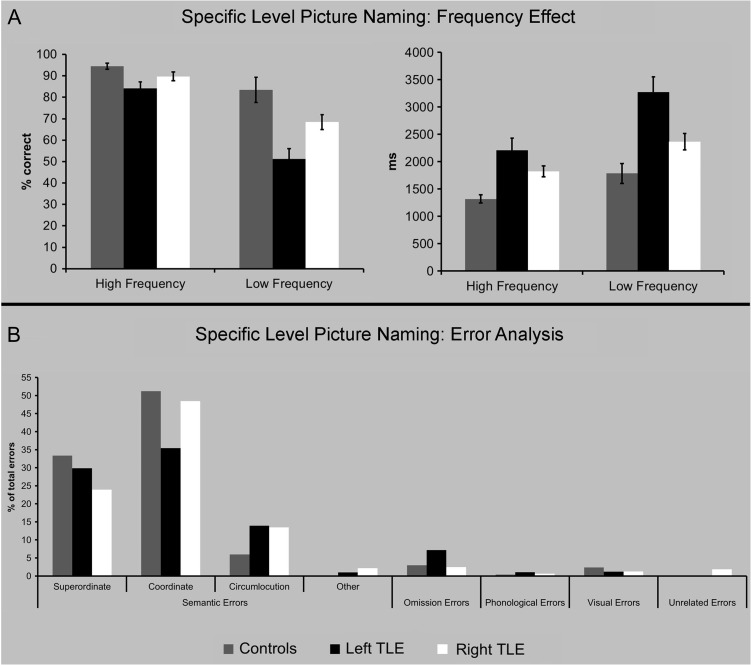
Specific-level picture naming results. (*A*) Picture naming results for high and low frequency items across the 3 groups for accuracy (left) and reaction time (right). Error bars show standard error. (*B*) Results from the error analysis are separated by error category.

In line with previous results that the left ATL displays some specialization for speech production tasks, left TLE patients were slower and less accurate than right TLE patients and control participants in naming specific-level items. Right TLE patients were also slower compared with control participants but not to the extent of the left TLE group (Fig. 2, Supplementary Table S1). Further inspection of the data revealed that greater word-finding difficulties in the patients was driven by the lower frequency items (Fig. 4). Two-way mixed ANOVAs with main effects of Group (controls, left TLE, right TLE) and Frequency (high, low) were run on the accuracy and reaction time data separately. Significant Group × Frequency interactions (accuracy: *F*[2, 57] = 6.84, *P* = 0.002; reaction time: *F*[2, 57] = 4.15, *P* = 0.021) showed that all 3 groups were slower and less accurate when naming low frequency items, although this effect was strongest in the left TLE group.

The types of errors made across the 3 groups were examined (Fig. 4*B*), and incorrect responses were coded according to the following categories: semantic errors (superordinate, coordinate, circumlocutions, subordinate, “It’s not a… (correct)”), phonological errors (formal, mixed, initial phoneme), omission errors (no response, preseveration), visual errors, and unrelated errors. Although TLE patients made more errors than controls, their distribution over error types was similar. For all groups the majority of errors were semantic (superordinate e.g., “Labrador” → “dog”/coordinate, e.g., “leopard” → “cheetah”) or omissions.

### Famous Face Naming

Overall, this task was one of the most challenging in the battery for control participants, as well as the TLE patients; as a result the reaction times were long and highly variable across participants, so our analyses are restricted to accuracy. The accuracy data were analyzed in the same way as in Drane et al. (2012) but with all items included in the analysis (although very similar results were obtained when we excluded items unfamiliar to participants; see Supplementary Fig. S2). Again, in line with the proposal that the left ATL has some specialization for speech production tasks, left TLE patients were less accurate at naming people compared with control participants and right TLE patients. Right TLE patients were also less accurate compared with control participants (Fig. 2, Supplementary Table S1). This mirrors the performance of patients on the picture naming task described above. In contrast to the prediction that the right ATL has some specialization for visual recognition (Drane et al. 2012), we found that both patient groups were equally impaired at recognizing famous faces compared with control participants (Fig. 2, Supplementary Table S1).

### Face-Name and Face-Description Matching

In contrast to the relative weakness of left TLE patients on the expressive face naming task, in terms of accuracy on the Face-Name matching test both groups of TLE patients showed a general weakness compared with controls. This pattern was also replicated in the reaction time data (Fig. 2, Supplementary Table S1). In the Face-Description matching test, again there was no evidence for a relative weakness in the right TLE group for recognition of famous faces; instead both patient groups were slower and less accurate compared with control participants (Fig. 2, Supplementary Table S1).

### Face Familiarity

There was a greater weakness in the right TLE group compared with the left TLE group and controls in terms of accuracy. Both TLE groups showed slower responses compared with control participants (Fig. 2, Supplementary Table S1).

### Unfamiliar Face Perception

Here, in line with the results reported so far, both patient groups showed less accurate responses on the Glasgow face matching task compared with control participants. The right TLE group also showed a greater weakness in terms of response time compared with controls (Fig. 2, Supplementary Table S1).

### Emotion Recognition

For brevity only the 100% morphed image results are reported here. Fitting with the overall findings reported above, both patient groups showed weaker performance on the emotion recognition task compared with controls in terms of accuracy and reaction time (Fig. 2, Supplementary Table S1). This finding was mirrored across all emotion categories both in terms of accuracy and reaction time (see Supplementary Materials, Supplementary Fig. S3 for further analysis).

### Camel and Cactus Test

The results from the verbal and nonverbal versions of the Camel and Cactus test are shown in Table 3. As with the battery as a whole, both the left and right TLE patients were slower on the word and picture versions of the test compared with control participants (written words: *F*[2, 49] = 22.17, *P* < 0.0001, left TLE vs. controls: *t*[49] = 5.20, *P* < 0.0001; right TLE vs. controls: *t*[49] = 6.04, *P* < 0.0001; pictures: *F*[2, 49] = 26.27, *P* < 0.0001; left TLE vs. controls: *t*[49] = 5.57, *P* < 0.0001; right TLE vs. controls: *t*[49] = 6.63, *P* < 0.0001).There was no difference between the patient groups in terms of reaction time. The left TLE group did show poorer accuracy on the word version of the test compared with the control group only (*F*[2, 50] = 3.71, *P* = 0.03; left TLE vs. controls: *t*[50] = 2.53, *P* = 0.015). The corresponding difference between the right TLE and controls on the picture version was not significant (*F*[2, 50] = 0.52, *P* = 0.60).

**Table 3:** Camel and Cactus behavioral performance. Data reported here are on a subset of TLE patients who took part in a second experimental study. Mean accuracy (%) and correct response times (ms) across the 2 versions of the Camel and Cactus test (standard deviation in parenthesis).

	Written Word	Picture
% Correct		
Left TLE (*n* = 16)	88 (7)^a^	78 (7)
Right TLE (*n* = 17)	91 (6)	79 (6)
Controls (*n* = 20)	94 (5)	81 (6)
Correct response time (ms)		
Left TLE (*n* = 15)	2387 (378)^a^	2427 (344)^a^
Right TLE (*n* = 17)	2396 (340)^a^	2520 (323)^a^
Controls (*n* = 20)	1714 (303)	1811 (262)

^a^Significant difference between the TLE group versus controls. No patient group comparisons were significant

#### Effect of Surgical Resection Volume on Behavioral Performance

The resection surgery for the patient cohort reported here was consistent across individuals; however, there were group differences in the volume of tissue resected. To explore the potential impact of resection volume, we ran ANCOVAs on the patient data (left TLE: *n* = 18; right TLE: *n* = 17) for whom we had a measure of resection volume. There was limited change in the results reported above for the full patient group (see Supplementary Table S2). The only instance where resection volume was significantly correlated with behavioral performance and changed the main effect (left vs. right) was for accuracy performance on the face-description matching test: before accounting for resection volume there was no group difference between the left and right TLE patients (*F*[1, 33] = 0.09, *P* = 0.76), however, after accounting for this there was a marginal effect driven by the left TLE group performing less accurately than the right TLE group (Group: *F*[2, 32] = 2.33, *P* = 0.14; Covariate of resection volume: *F*[1, 32] = 4.14, *P* = 0.05).

## Discussion

This investigation comprehensively mapped the status of verbal and nonverbal semantic processing and related abilities in TLE patients with unilateral left versus right ATL resection. The results are of both clinical and theoretical interest given the debates regarding the individual contributions of the left and right ATLs to conceptual knowledge (Damasio et al. 2004; Olson et al. 2007, 2013; Drane et al. 2012; Gainotti 2012, 2014; Rice, Lambon Ralph, et al. 2015). Using a systematic, detailed battery of semantic tests, the principal findings were of a mild semantic impairment in left and right resected patients, and that the semantic impairments were heightened during challenging semantic processing (e.g., on low frequency/imageability items). Graded differences in performance between the patient groups were secondary to the overall mild semantic impairment; primarily, left TLE patients showed weaker performance on tasks that required naming or accessing semantic information from a written word. In addition, right TLE patients showed weaker performance in face recognition, though this effect was observed less consistently across tasks. These neuropsychological data fit closely with the data arising from both other patient groups and results from rTMS and fMRI in healthy participants. Together these convergent, multimethod data strongly point towards the left and right ATL making similar, parallel contributions to a singular functional system for semantic representation.

It has become increasingly clear that bilateral ATL damage causes severe semantic impairments whereas, comparatively, unilateral ATL damage/resection produces relatively mild semantic impairments in both humans (Terzian and Ore 1955 ; Scoville and Milner 1957; Lambon Ralph et al. 2010, 2012) and animals (Brown and Schafer 1888; Kluver and Bucy 1937, 1939). For example, the TLE patients studied by Lambon Ralph et al. (2012) averaged 85% accuracy in specific-level picture naming (cf. control performance >95%), whereas scores of 20% or less are common in SD patients on the same task (Rogers et al. 2015). This suggests that degradation of semantic representations following unilateral resection is milder than that seen in SD patients but is still sufficient to reduce the efficiency of semantic processing (Wilkins and Moscovitch 1978; Lambon Ralph et al. 2010, 2012). Thus, semantic impairments may only be apparent in unilateral patients on more demanding assessments. The marked difference in the severity of semantic impairment following unilateral (TLE) versus bilateral (SD) damage, in additional to evidence of bilateral activation in fMRI studies and equivalent effects of left or right ATL TMS, led Lambon Ralph et al. (2012) to suggest that conceptual knowledge is represented in a distributed fashion across the ATLs, bilaterally (see also, Kluver and Bucy 1938; Terzian and Ore 1955; Lambon Ralph et al. 2010; Rice, Lambon Ralph, et al. 2015). This bilateral representation of conceptual knowledge affords the semantic system a degree of redundancy and makes it somewhat resistant to unilateral damage (Schapiro et al. 2013). Indeed this hypothesis has been formally explored and supported both through formal computational modeling and combined TMS–fMRI explorations in healthy participants (Schapiro et al. 2013; Binney and Lambon Ralph 2015; Jung and Lambon Ralph 2016).

The mild semantic impairment observed in TLE patients was amplified during more challenging semantic processing. In healthy controls, response times increased when low frequency and low imageability concepts were probed. In the TLE patients, this effect was exaggerated and accuracy also worsened under these conditions. Similar effects of frequency and imageability have been observed in SD patients (Jefferies et al. 2009; Hoffman and Lambon Ralph 2011; Rogers et al. 2015) and appear to reflect the intrinsic weakness of low frequency/imageability concepts. Less imageable concepts not only have impoverished semantic representations (because they do not benefit from sensory-motor associations) but they also have more context-dependent meanings (Hoffman 2016). Likewise, low frequency items are less strongly represented because the semantic system has fewer opportunities to acquire their meanings (Rogers and McClelland 2004). It is important to note that frequency effects are not observed in all patient groups with semantic impairment (cf. patients with executive-semantic impairments rather than degradation of semantic representations per se show smaller frequency effects on some tasks: Jefferies and Lambon Ralph 2006; Hoffman et al. 2011; Rogers et al. 2015). This result therefore indicates that TLE patients differ from SD patients in the severity of their semantic impairment but not in the general nature of the impairment. Indeed, on the somewhat rare occasions that SD patients present clinically in the very early stages (Bozeat et al. 2000; Adlam et al. 2006) their semantic profiles are close to those observed here for resected TLE patients.

Secondary to the parallel semantic impairments found across both TLE groups (consistent with the convergent clinical and cognitive neuroscience data that both the left and right ATL primarily make the same contribution to a singular functional system for semantic representation), subtle graded differences in performance between the left and right TLE groups were also observed. The most robust functional gradation between the groups was observed in the naming tasks, where the left TLE group showed a marked deficit in accuracy (mainly through omission errors) as well as reaction time. This is in accordance with the well-established hypothesis that the left ATL has a more prominent role in word retrieval (Lambon Ralph et al. 2001; Damasio et al. 2004) and aligns with repeated observations of word finding difficulties after left ATL damage/resection (Bi et al. 2011; Drane et al. 2012). The right TLE group also produced slower and less accurate naming responses compared with the control group, but not to the same level of the left TLE group, suggesting that this hemispheric specialization is graded rather than absolute. Differences between the 2 patient groups were also shown on tasks involving access to semantic information from written words (e.g., synonym judgment, lexical decision). This provides partial support for the input modality hypothesis (Gainotti 2012), which suggests that the left ATL is relatively specialized for processing verbal inputs (e.g., written and spoken words), and also aligns with a wider literature showing a greater degree of impairment on verbal short-term memory after left ATL resection (Saling 2009; Willment and Golby 2013). There was less consistent evidence, however, for the corresponding effect of more impaired processing of nonverbal information in right TLE.

The finding of relatively poorer performance during naming/written word processing after left ATL resection is in accordance with the findings from neurologically intact participants (Rice, Lambon Ralph, et al. 2015). This meta-analysis of fMRI studies in healthy participants showed that semantic tasks elicited bilateral ATL activation, with the exception of naming and written word processing, which showed an increased likelihood of activation in the left ATL (Rice, Lambon Ralph, et al. 2015). Taken together, the patient results and fMRI data are consistent with previous proposals and findings that semantic processing is underpinned by a bilateral, yet graded system of semantic representation, whereby both the left and right ATLs are critical for normal semantic processing, yet secondary gradations in function can occur between (and within) the ATLs based on differential functional and structural connectivity (Lambon Ralph et al. 2001; Binney et al. 2012; Schapiro et al. 2013; Rice, Lambon Ralph, et al. 2015). The relative impairments in the verbal domain after left ATL resection may be driven by differential connectivity between the ATL and extra temporal language areas (Binney et al. 2012; Hurley et al. 2015; Rice, Hoffman, et al. 2015; Rice, Lambon Ralph, et al. 2015). In particular, the speech production system in the prefrontal cortex has been shown to be highly left lateralized in neuroimaging studies (Blank et al. 2002). Since intrahemispheric connections are much stronger than interhemispheric connections, particularly where language functions are concerned (Friederici et al. 2011; Herve et al. 2013), it follows that the left ATL is more strongly connected to regions involved in speech production than the right, and thus is likely to take on some specialization for naming tasks. This principle has been demonstrated formally by neuroanatomically constrained computational models (Lambon Ralph et al. 2001; Schapiro et al. 2013).

In contrast to the relative impairments in the verbal domain for the left TLE group, corresponding selective deficits in right TLE patients were less common. In the literature, the right ATL has been proposed to be crucial for visual recognition (Damasio et al. 2004, 2012), social cognition (Olson et al. 2013), or for emotional concepts (Monti and Meletti 2015). To explore these possibilities simultaneously within a single sample of TLE patients, we included tests of famous face and emotion recognition (Drane et al. 2012; Sedda et al. 2013) as a part of the much larger, detailed neuropsychological battery. In contrast to these proposals, we found a striking impairment in both patient groups compared with the control participants. Face and emotion recognition in both the left and right TLE groups was equally impaired. The lack of relative impairments for the right TLE group in these domains suggests that these tasks may instead rely on a bilateral ATL representation of conceptual knowledge. Similar conclusions were reached in a recent meta-analysis of emotion recognition in TLE. Specifically, the authors noted that evidence for laterality effects were conflicting and remained a key area for future research (Monti and Meletti 2015). Our suggestion that face and emotion recognition may rely on a bilateral ATL representation also fits with the results from a previous fMRI meta-analysis, which showed that visual semantic and social concept tasks exhibit strong bilateral activation across the ATLs in healthy participants (Rice, Lambon Ralph, et al. 2015), as well as a recent TMS study which showed impaired social concept processing after left or right ATL TMS (Pobric et al. 2016).

At first glance, the findings from the TLE patients and parallel results from healthy participants might seem at variance with some data reported for SD/FTD patients with asymmetric ATL atrophy. With regards to verbal and nonverbal versions of the same semantic task, performance differences have been observed between left > right and right > left patient groups (Snowden et al. 2012, 2017; Luzzi et al. 2017)—though it should be noted that the patients were not matched for the degree of overall ATL atrophy. Likewise, the finding of emotion recognition deficits in both left and right TLE groups without any relative differences might appear to be at odds with studies of emotion processing in the FTD literature (Chan et al. 2009; Binney et al. 2016). In this literature a number of studies report more impaired processing in right > left ATL atrophy groups compared with those with left > right ATL atrophy (Perry et al. 2001; Rosen et al. 2002; Seeley et al. 2005; Binney et al. 2016; Luzzi et al. 2017; Multani et al. 2017). However, the presence of bilateral (albeit asymmetric) temporal lobe atrophy and some degree of concurrent orbitofrontal lobe atrophy (even in SD with predominantly ATL > orbitofrontal atrophy), means that it is not straightforward to compare the results from TLE and FTD. In addition, in most of the studies comparing left > right and right > left atrophy, the latter group commonly have more temporal and frontal atrophy overall (Chan et al. 2009). Indeed, even when patients have been matched pairwise for the total temporal lobe atrophy, disparities in orbitofrontal atrophy remain (cf. Seeley et al. 2005). Accordingly, it would be useful for future studies to consider the impact of total temporal or frontotemporal atrophy on task performance alongside the laterality of pathology (as conducted by Luzzi et al. 2017 which showed that voice and face matching were correlated with right temporal FDG levels even after accounting for bilateral temporal FDG, which also correlated with task performance).

One remaining question is why some studies report localized functions in the right ATL for famous face recognition (Ellis et al. 1989; Drane et al. 2008, 2012; Olson et al. 2015). One possible explanation could be that the methods used to test the function of interest were not sensitive enough and thus the impairment in the left ATL group was missed. As noted in the current study on all the general semantic tasks, semantic impairments in resected TLE cases are relatively subtle and require more demanding, sensitive measures if they are to be reliably detected (Wilkins and Moscovitch 1978; Lambon Ralph et al. 2012).

To finish, it is important to note that the reported findings necessarily reflect postsurgical behavioral performance. Accordingly, no conclusions can be drawn regarding (1) presurgical performance and (2) the specific effect of surgery, that is, whether behavioral performance has improved/declined as a result of the resection surgery. Both factors have been reported to be important in understanding the cognitive profile of TLE. Changes in cognitive performance have been reported before resection surgery (Cheung et al. 2009; Helmstaedter and Elger 2009; Bonelli et al. 2012). Hermann et al. (2007) report a gradation in cognitive performance presurgery ranging from patients who exhibit no cognitive impairments presurgery, whereas others exhibit relatively selective memory impairment and a third group showed a generalized impairment which affects memory, processing speed and executive functioning (Hermann et al. 2007). As previously stated all patients included in this study have a long history of epilepsy and hippocampal sclerosis which leaves open the possibility of atypical reorganization of function. Accordingly, an important area of future research will be to compare presurgery versus postsurgery semantic performance.

## Supplementary Material

Supplementary Data

## References

[bhx362C95] Acres K, Taylor KI, Moss HE, Stamatakis EA, Tyler LK 2009 Complementary hemispheric asymmetries in object naming and recognition: A voxel-based correlational study. Neuropsychologia 47(8-9):1836–1843. 19428415 10.1016/j.neuropsychologia.2009.02.024

[bhx362C1] Adlam ALR, Patterson K, Rogers TT, Nestor PJ, Salmond CH, Acosta-Cabronero J, Hodges JR 2006 Semantic dementia and fluent primary progressive aphasia: two sides of the same coin? Brain. 129:3066–3080. 17071925 10.1093/brain/awl285

[bhx362C2] Bi Y, Wei T, Wu C, Han Z, Jiang T, Caramazza A 2011 The role of the left anterior temporal lobe in language processing revisited: evidence from an individual with ATL resection. Cortex. 47(5):575–587. 20074721 10.1016/j.cortex.2009.12.002

[bhx362C3] Binney RJ, Embleton KV, Jefferies E, Parker GJ, Lambon Ralph MA 2010 The ventral and inferolateral aspects of the anterior temporal lobe are crucial in semantic memory: evidence from a novel direct comparison of distortion-corrected fMRI, rTMS, and semantic dementia. Cereb Cortex. 20(11):2728–2738. 20190005 10.1093/cercor/bhq019

[bhx362C4] Binney RJ, Henry ML, Babiak M, Pressman PS, Santos-Santos MA, Narvid J, Mandelli ML, Strain PJ, Miller BL, Rankin KP, et al 2016 Reading words and other people: a comparison of exception word, familiar face and affect processing in the left and right temporal variants of primary progressive aphasia. Cortex. 82:147–163. 27389800 10.1016/j.cortex.2016.05.014 PMC4969161

[bhx362C5] Binney RJ, Lambon Ralph MA 2015 Using a combination of fMRI and anterior temporal lobe rTMS to measure intrinsic and induced activation changes across the semantic cognition network. Neuropsychologia. 76:170–181. 25448851 10.1016/j.neuropsychologia.2014.11.009 PMC4582802

[bhx362C6] Binney RJ, Parker GJ, Lambon Ralph MA 2012 Convergent connectivity and graded specialization in the rostral human temporal lobe as revealed by diffusion-weighted imaging probabilistic tractography. J Cogn Neurosci. 24(10):1998–2014. 22721379 10.1162/jocn_a_00263

[bhx362C7] Blank SC, Scott SK, Murphy K, Warburton E, Wise RJS 2002 Speech production: Wernicke, Broca and beyond. Brain. 125:1829–1838. 12135973 10.1093/brain/awf191

[bhx362C8] Bonelli SB, Thompson PJ, Yogarajah M, Vollmar C, Powell RHW, Symms MR, McEvoy AW, Micallef C, Koepp MJ, Duncan JS 2012 Imaging language networks before and after anterior temporal lobe resection: results of a longitudinal fMRI study. Epilepsia. 53(4):639–650. 22429073 10.1111/j.1528-1167.2012.03433.x PMC4471632

[bhx362C9] Bora E, Meletti S 2016 Social cognition in temporal lobe epilepsy: a systematic review and meta-analysis. Epilepsy Behav. 60:50–57. 27179192 10.1016/j.yebeh.2016.04.024

[bhx362C10] Bozeat S, Lambon Ralph MA, Patterson K, Garrard P, Hodges JR 2000 Non-verbal semantic impairment in semantic dementia. Neuropsychologia. 38(9):1207–1215. 10865096 10.1016/s0028-3932(00)00034-8

[bhx362C11] Brown S, Schafer EA 1888 An investigation into the functions of the occipital and temporal lobes of the monkey’s brain. Philos Trans R Soc Lond B Biol Sci. 179:303–327.

[bhx362C12] Burton AM, White D, McNeill A 2010 The Glasgow face matching test. Behav Res Methods. 42(1):286–291. 20160307 10.3758/BRM.42.1.286

[bhx362C13] Chan D, Anderson V, Pijnenburg Y, Whitwell J, Barnes J, Scahill R, Stevens JM, Barkhof F, Scheltens P, Rossor MN, et al 2009 The clinical profile of right temporal lobe atrophy. Brain. 132:1287–1298. 19297506 10.1093/brain/awp037

[bhx362C14] Cheung MC, Chan AS, Lam JMK, Chan YL 2009 Pre- and postoperative fMRI and clinical memory performance in temporal lobe epilepsy. J Neurol Neurosurg Psychiatry. 80(10):1099–1106. 19389718 10.1136/jnnp.2009.173161

[bhx362C15] Damasio H, Tranel D, Grabowski T, Adolphs R, Damasio A 2004 Neural systems behind word and concept retrieval. Cognition. 92(1–2):179–229. 15037130 10.1016/j.cognition.2002.07.001

[bhx362C16] Drane DL, Ojemann GA, Aylward E, Ojemann JG, Johnson LC, Silbergeld DL, Miller JW, Tranel D 2008 Category-specific naming and recognition deficits in temporal lobe epilepsy surgical patients. Neuropsychologia. 46(5):1242–1255. 18206185 10.1016/j.neuropsychologia.2007.11.034 PMC2474808

[bhx362C17] Drane DL, Ojemann JG, Phatak V, Loring DW, Gross RE, Hebb AO, Silbergeld DL, Miller JW, Voets NL, Saindane AM, et al 2012 Famous face identification in temporal lobe epilepsy: support for a multimodal integration model of semantic memory. Cortex. 49(6):1648–1667. 23040175 10.1016/j.cortex.2012.08.009 PMC3679345

[bhx362C18] Edwards-Lee T, Miller BL, Benson DF, Cummings JL, Russell GL, Boone K, Mena I 1997 The temporal variant of frontotemporal dementia. Brain. 120(Pt 6):1027–1040. 9217686 10.1093/brain/120.6.1027

[bhx362C19] Ellis AW, Young AW, Critchley EM 1989 Loss of memory for people following temporal lobe damage. Brain. 112(Pt 6):1469–1483. 2597991 10.1093/brain/112.6.1469

[bhx362C20] Friederici AD, Brauer J, Lohmann G 2011 Maturation of the language network: from inter- to intrahemispheric connectivities. PLoS One. 6(6):e20726. 21695183 10.1371/journal.pone.0020726 PMC3113799

[bhx362C21] Gainotti G 2007 Different patterns of famous people recognition disorders in patients with right and left anterior temporal lesions: a systematic review. Neuropsychologia. 45(8):1591–1607. 17275042 10.1016/j.neuropsychologia.2006.12.013

[bhx362C22] Gainotti G 2012 The format of conceptual representations disrupted in semantic dementia: a position paper. Cortex. 48(5):521–529. 21807363 10.1016/j.cortex.2011.06.019

[bhx362C23] Gainotti G 2014 Why are the right and left hemisphere conceptual representations different? Behav Neurol. 2014:603134. 24803728 10.1155/2014/603134 PMC4006601

[bhx362C24] Gallate J, Wong C, Ellwood S, Chi R, Snyder A 2011 Noninvasive brain stimulation reduces prejudice scores on an implicit association test. Neuropsychology. 25(2):185–192. 21381825 10.1037/a0021102

[bhx362C25] Galton CJ, Patterson K, Graham K, Lambon Ralph MA, Williams G, Antoun N, Sahakian BJ, Hodges JR 2001 Differing patterns of temporal atrophy in Alzheimer’s disease and semantic dementia. Neurology. 57(2):216–225. 11468305 10.1212/wnl.57.2.216

[bhx362C26] Geschwind N 1979 Behavioural changes in temporal lobe epilepsy. Psychol Med. 9(2):217–219. 472070 10.1017/s0033291700030713

[bhx362C27] Glosser G, Salvucci AE, Chiaravalloti ND 2003 Naming and recognizing famous faces in temporal lobe epilepsy. Neurology. 61(1):81–86. 12847161 10.1212/01.wnl.0000073621.18013.e1

[bhx362C28] Helmstaedter C, Elger CE 2009 Chronic temporal lobe epilepsy: a neurodevelopmental or progressively dementing disease? Brain. 132:2822–2830. 19635728 10.1093/brain/awp182

[bhx362C29] Hermann B, Seidenberg M, Lee E-J, Chan F, Rutecki PA 2007 Cognitive phenotypes in temporal lobe epilepsy. J Int Neuropsychol Soc. 13:12–20. 17166299 10.1017/S135561770707004X

[bhx362C30] Herve PY, Zago L, Petit L, Mazoyer B, Tzourio-Mazoyer N 2013 Revisiting human hemispheric specialization with neuroimaging. Trends Cogn Sci. 17(2):69–80. 23317751 10.1016/j.tics.2012.12.004

[bhx362C31] Hoffman P 2016 The meaning of ‘life’ and other abstract words: insights from neuropsychology. J Neuropsychol. 10(2):317–343. 25708527 10.1111/jnp.12065 PMC5026063

[bhx362C32] Hoffman P, Lambon Ralph MA 2011 Reverse concreteness effects are not a typical feature of semantic dementia: evidence for the hub-and-spoke model of conceptual representation. Cereb Cortex. 21(9):2103–2112. 21285258 10.1093/cercor/bhq288

[bhx362C33] Hoffman P, Rogers TT, Lambon Ralph MA 2011 Semantic diversity accounts for the “missing” word frequency effect in stroke aphasia: insights using a novel method to quantify contextual variability in meaning. J Cogn Neurosci. 23(9):2432–2446. 21254804 10.1162/jocn.2011.21614

[bhx362C34] Hurley RS, Bonakdarpour B, Wang X, Mesulam MM 2015 Asymmetric connectivity between the anterior temporal lobe and the language network. J Cogn Neurosci. 27(3):464–473. 25244113 10.1162/jocn_a_00722 PMC4312550

[bhx362C35] Jefferies E, Lambon Ralph MA 2006 Semantic impairment in stroke aphasia versus semantic dementia: a case-series comparison. Brain. 129(Pt 8):2132–2147. 16815878 10.1093/brain/awl153

[bhx362C36] Jefferies E, Patterson K, Jones RW, Lambon Ralph MA 2009 Comprehension of concrete and abstract words in semantic dementia. Neuropsychology. 23(4):492–499. 19586212 10.1037/a0015452 PMC2801065

[bhx362C37] Jung J, Lambon Ralph MA 2016 Mapping the dynamic network interactions underpinning cognition: a cTBS-fMRI study of the flexible adaptive neural system for semantics. Cereb Cortex. 26:3580–3590. 27242027 10.1093/cercor/bhw149 PMC4961025

[bhx362C38] Kluver H, Bucy PC 1937 “Psychic blindness” and other symptoms following bilateral temporal lobectomy in rhesus monkeys. Am J Physiol. 119:352.

[bhx362C39] Kluver H, Bucy PC 1938 An analysis of certain effects of bilateral temporal lobectomy in the rhesus monkey, with special reference to “Psychic Blindness”. J Psychol. 5(1):33–54.

[bhx362C40] Kluver H, Bucy PC 1939 Preliminary analysis of functions of the temporal lobes in monkeys. Arch Neurol Psychiatry. 42(6):979–1000. 10.1176/jnp.9.4.606 9447506

[bhx362C41] Kumfor F, Landin-Romero R, Devenney E, Hutchings R, Grasso R, Hodges JR, Piguet O 2016 On the right side? A longitudinal study of left- versus right-lateralized semantic dementia. Brain. 139:986–998. 26811253 10.1093/brain/awv387

[bhx362C42] Lambon Ralph MA 2014 Neurocognitive insights on conceptual knowledge and its breakdown. Philos Trans R Soc Lond B Biol Sci. 369:1–12. 10.1098/rstb.2012.0392 PMC3866422 24324236

[bhx362C43] Lambon Ralph MA, Cipolotti L, Manes F, Patterson K 2010 Taking both sides: do unilateral anterior temporal lobe lesions disrupt semantic memory? Brain. 133(11):3243–3255. 20952378 10.1093/brain/awq264

[bhx362C44] Lambon Ralph MA, Ehsan S, Baker GA, Rogers TT 2012 Semantic memory is impaired in patients with unilateral anterior temporal lobe resection for temporal lobe epilepsy. Brain. 135(Pt 1):242–258. 22287382 10.1093/brain/awr325 PMC3267985

[bhx362C45] Lambon Ralph MA, Jefferies E, Patterson K, Rogers TT 2017 The neural and computational bases of semantic cognition. Nat Rev Neurosci. 18(1):42–55. 27881854 10.1038/nrn.2016.150

[bhx362C46] Lambon Ralph MA, McClelland JL, Patterson K, Galton CJ, Hodges JR 2001 No right to speak? The relationship between object naming and semantic impairment: neuropsychological evidence and a computational model. J Cogn Neurosci. 13(3):341–356. 11371312 10.1162/08989290151137395

[bhx362C47] Luzzi S, Baldinelli S, Ranaldi V, Fabi K, Cafazzo V, Fringuelli F, Silvestrini M, Provinciali L, Reverberi C, Gainotti G 2017 Famous faces and voices: differential profiles in early right and left semantic dementia and in Alzheimer’s disease. Neuropsychologia. 94:118–128. 27916672 10.1016/j.neuropsychologia.2016.11.020

[bhx362C48] Mesulam MM, Wieneke C, Hurley R, Rademaker A, Thompson CK, Weintraub S, Rogalski EJ 2013 Words and objects at the tip of the left temporal lobe in primary progressive aphasia. Brain. 136:601–618. 23361063 10.1093/brain/aws336 PMC3572925

[bhx362C49] Mion M, Patterson K, Acosta-Cabronero J, Pengas G, Izquierdo-Garcia D, Hong YT, Fryer TD, Williams GB, Hodges JR, Nestor PJ 2010 What the left and right anterior fusiform gyri tell us about semantic memory. Brain. 133(11):3256–3268. 20952377 10.1093/brain/awq272

[bhx362C50] Moll J, Zahn R, de Oliveira-Souza R, Krueger F, Grafman J 2005 The neural basis of human moral cognition. Nat Rev Neurosci. 6(10):799–809. 16276356 10.1038/nrn1768

[bhx362C51] Monti G, Meletti S 2015 Emotion recognition in temporal lobe epilepsy: a systematic review. Neurosci Biobehav Rev. 55:280–293. 25999121 10.1016/j.neubiorev.2015.05.009

[bhx362C52] Multani N, Galantucci S, Wilson SM, Shany-Ur T, Poorzand P, Growdon ME, Jang JY, Kramer JH, Miller BL, Rankin KP, et al 2017 Emotion detection deficits and changes in personality traits linked to loss of white matter integrity in primary progressive aphasia. Neuroimage Clin. 16:447–454. 28879086 10.1016/j.nicl.2017.08.020 PMC5577436

[bhx362C53] Mummery CJ, Patterson K, Price CJ, Ashburner J, Frackowiak RS, Hodges JR 2000 A voxel-based morphometry study of semantic dementia: relationship between temporal lobe atrophy and semantic memory. Ann Neurol. 47(1):36–45. 10632099

[bhx362C54] Olson IR, Ezzyat Y, Plotzker A, Chatterjee A 2015 The end point of the ventral visual stream: face and non-face perceptual deficits following unilateral anterior temporal lobe damage. Neurocase. 21(5):554–562. 25238048 10.1080/13554794.2014.959025 PMC4366355

[bhx362C55] Olson IR, McCoy D, Klobusicky E, Ross LA 2013 Social cognition and the anterior temporal lobes: a review and theoretical framework. Soc Cogn Affect Neurosci. 8(2):123–133. 23051902 10.1093/scan/nss119 PMC3575728

[bhx362C56] Olson IR, Ploaker A, Ezzyat Y 2007 The Enigmatic temporal pole: a review of findings on social and emotional processing. Brain. 130:1718–1731. 17392317 10.1093/brain/awm052

[bhx362C57] Osterrieth P-A 1944 The challenge of copying a complex figure. Arch Psychol. 30(117–20):205–353.

[bhx362C58] Patterson K, Lambon Ralph MA, Jefferies E, Woollams A, Jones R, Hodges JR, Rogers TT 2006 “Presemantic” cognition in semantic dementia: six deficits in search of an explanation. J Cogn Neurosci. 18(2):169–183. 16494679 10.1162/089892906775783714

[bhx362C59] Perry RJ, Rosen HR, Kramer JH, Beer JS, Levenson RL, Miller BL 2001 Hemispheric dominance for emotions, empathy and social behaviour: evidence from right and left handers with frontotemporal dementia. Neurocase. 7(2):145–160. 11320162 10.1093/neucas/7.2.145

[bhx362C60] Pobric G, Jefferies E, Lambon Ralph MA 2007 Anterior temporal lobes mediate semantic representation: mimicking semantic dementia by using rTMS in normal participants. Proc Natl Acad Sci USA. 104(50):20137–20141. 18056637 10.1073/pnas.0707383104 PMC2148435

[bhx362C61] Pobric G, Jefferies E, Lambon Ralph MA 2010 Amodal semantic representations depend on both anterior temporal lobes: evidence from repetitive transcranial magnetic stimulation. Neuropsychologia. 48(5):1336–1342. 20038436 10.1016/j.neuropsychologia.2009.12.036

[bhx362C62] Pobric G, Lambon Ralph MA, Zahn R 2016 Hemispheric specialization within the superior anterior temporal cortex for social and nonsocial concepts. J Cogn Neurosci. 28(3):351–360. 26544918 10.1162/jocn_a_00902

[bhx362C63] Rice GE, Hoffman P, Lambon Ralph MA 2015 Graded specialization within and between the anterior temporal lobes. Ann NY Acad Sci USA. 1359:84–97. 10.1111/nyas.12951 PMC4982095 26502375

[bhx362C64] Rice GE, Lambon Ralph MA, Hoffman P 2015 The roles of the left vs. right anterior temporal lobes in conceptual knowledge: an ALE meta-analysis of 97 functional neuroimaging studies. Cereb Cortex. 25(11):4374–4391. 25771223 10.1093/cercor/bhv024 PMC4816787

[bhx362C65] Rogers TT, Lambon Ralph MA, Hodges JR, Patterson K 2004 Natural selection: the impact of semantic impairment on lexical and object decision. Cogn Neuropsychol. 21(2–4):331–352. 21038209 10.1080/02643290342000366

[bhx362C66] Rogers TT, McClelland JL 2004 Semantic cognition: a parallel distributed processing approach. Cambridge, MA: MIT Press. 10.1038/nrn1076 12671647

[bhx362C67] Rogers TT, Patterson K, Jefferies E, Lambon Ralph MA 2015 Disorders of representation and control in semantic cognition: effects of familiarity, typicality and specificity. Neuropsychologia. 76:220–239. 25934635 10.1016/j.neuropsychologia.2015.04.015 PMC4582808

[bhx362C68] Rosen HJ, Perry RJ, Murphy J, Kramer JH, Mychack P, Schuff N, Weiner M, Levenson RW, Miller BL 2002 Emotion comprehension in the temporal variant of frontotemporal dementia. Brain. 125:2286–2295. 12244085 10.1093/brain/awf225

[bhx362C69] Saling MM 2009 Verbal memory in mesial temporal lobe epilepsy: beyond material specificity. Brain. 132:570–582. 19251757 10.1093/brain/awp012

[bhx362C70] Schapiro AC, McClelland JL, Welbourne SR, Rogers TT, Lambon Ralph MA 2013 Why bilateral damage is worse than unilateral damage to the brain. J Cogn Neurosci. 25(12):2107–2123. 23806177 10.1162/jocn_a_00441

[bhx362C71] Scoville WB, Milner B 1957 Loss of recent memory after bilateral hippocampal lesions. J Neurol Neurosurg Psychiatry. 20(1):11–21. 13406589 10.1136/jnnp.20.1.11 PMC497229

[bhx362C72] Sedda A, Rivolta D, Scarpa P, Burt M, Frigerio E, Zanardi G, Piazzini A, Turner K, Canevini MP, Francione S, et al 2013 Ambiguous emotion recognition in temporal lobe epilepsy: the role of expression intensity. Cogn Affect Behav Neurosci. 13(3):452–463. 23430725 10.3758/s13415-013-0153-y

[bhx362C73] Seeley WW, Bauer AM, Miller BL, Gorno-Tempini ML, Kramer JH, Weiner M, Rosen HJ 2005 The natural history of temporal variant frontotemporal dementia. Neurology. 64(8):1384–1390. 15851728 10.1212/01.WNL.0000158425.46019.5C PMC2376750

[bhx362C74] Seghier ML, Ramlackhansingh A, Crinion J, Leff AP, Price CJ 2008 Lesion identification using unified segmentation-normalisation models and fuzzy clustering. Neuroimage. 41(4):1253–1266. 18482850 10.1016/j.neuroimage.2008.03.028 PMC2724121

[bhx362C75] Seidenberg M, Griffith R, Sabsevitz D, Moran M, Haltiner A, Bell B, Swanson S, Hammeke T, Hermann B 2002 Recognition and identification of famous faces in patients with unilateral temporal lobe epilepsy. Neuropsychologia. 40(4):446–456. 11684177 10.1016/s0028-3932(01)00096-3

[bhx362C76] Shimotake A, Matsumoto R, Ueno T, Kunieda T, Saito S, Hoffman P, Kikuchi T, Fukuyama H, Miyamoto S, Takahashi R, et al 2014 Direct exploration of the role of the ventral anterior temporal lobe in semantic memory: cortical stimulation and local field potential evidence from subdural grid electrodes. Cereb Cortex. 25(10):3802–3817. 25491206 10.1093/cercor/bhu262 PMC4585516

[bhx362C77] Skipper LM, Ross LA, Olson IR 2011 Sensory and semantic category subdivisions within the anterior temporal lobes. Neuropsychologia. 49(12):3419–3429. 21889520 10.1016/j.neuropsychologia.2011.07.033 PMC3192293

[bhx362C78] Snowden JS, Goulding PJ, Neary D 1989 Semantic dementia: a form of circumscribed cerebral atrophy. Behav Neurol. 2(3):167–182.

[bhx362C79] Snowden JS, Harris JM, Thompson JC, Kobylecki C, Jones M, Richardson AM, Neary D 2017 Semantic dementia and the left and right temporal lobes. Cortex. doi:10.1016/j.cortex.2017.08.024. 10.1016/j.cortex.2017.08.024 28947063

[bhx362C80] Snowden JS, Thompson JC, Neary D 2004 Knowledge of famous faces and names in semantic dementia. Brain. 127(Pt 4):860–872. 14985259 10.1093/brain/awh099

[bhx362C81] Snowden JS, Thompson JC, Neary D 2012 Famous people knowledge and the right and left temporal lobes. Behav Neurol. 25(1):35–44. 22207421 10.3233/BEN-2012-0347 PMC5294234

[bhx362C82] Terzian H, Ore GD 1955 Syndrome of Kluver and Bucy reproduced in man by bilateral removal of the temporal lobes. Neurology. 5(6):373–380. 14383941 10.1212/wnl.5.6.373

[bhx362C83] Thompson SA, Patterson K, Hodges JR 2003 Left/right asymmetry of atrophy in semantic dementia: behavioral-cognitive implications. Neurology. 61(9):1196–1203. 14610120 10.1212/01.wnl.0000091868.28557.b8

[bhx362C84] Tranel D, Damasio H, Damasio AR 1997 A neural basis for the retrieval of conceptual knowledge. Neuropsychologia. 35(10):1319–1327. 9347478 10.1016/s0028-3932(97)00085-7

[bhx362C85] Visser M, Embleton KV, Jefferies E, Parker GJ, Ralph MAL 2010 The inferior, anterior temporal lobes and semantic memory clarified: novel evidence from distortion-corrected fMRI. Neuropsychologia. 48(6):1689–1696. 20176043 10.1016/j.neuropsychologia.2010.02.016

[bhx362C86] Visser M, Jefferies E, Embleton KV, Lambon Ralph MA 2012 Both the middle temporal gyrus and the ventral anterior temporal area are crucial for multimodal semantic processing: distortion-corrected fMRI evidence for a double gradient of information convergence in the temporal lobes. J Cogn Neurosci. 24(8):1766–1778. 22621260 10.1162/jocn_a_00244

[bhx362C87] Visser M, Lambon Ralph MA 2011 Differential contributions of bilateral ventral anterior temporal lobe and left anterior superior temporal gyrus to semantic processes. J Cogn Neurosci. 23(10):3121–3131. 21391767 10.1162/jocn_a_00007

[bhx362C88] Warrington EK 1996 The Camden memory tests manual. Hove: Psychology Press.

[bhx362C89] Wear HJ, Wedderburn CJ, Mioshi E, Williams-Gray CH, Mason SL, Barker RA, Hodges JR 2008 The Cambridge Behavioural Inventory revised. Dement Neuropsychol. 2(2):102–107. 29213551 10.1590/S1980-57642009DN20200005 PMC5619578

[bhx362C90] Wiebe S, Blume WT, Girvin JP, Eliasziw M, Effectiveness Efficiency Surgery T 2001 A randomized, controlled trial of surgery for temporal-lobe epilepsy. N Engl J Med. 345(5):311–318. 11484687 10.1056/NEJM200108023450501

[bhx362C91] Wilkins A, Moscovitch M 1978 Selective impairment of semantic memory afte temporaly lobectomy. Neuropsychologia. 16(1):73–79. 634464 10.1016/0028-3932(78)90044-1

[bhx362C92] Willment KC, Golby A 2013 Hemispheric lateralization interrupted: material-specific memory deficits in temporal lobe epilepsy. Front Hum Neurosci. 7:546. 24032014 10.3389/fnhum.2013.00546 PMC3759288

[bhx362C93] Zahn R, Moll J, Iyengar V, Huey ED, Tierney M, Krueger F, Grafman J 2009 Social conceptual impairments in frontotemporal lobar degeneration with right anterior temporal hypometabolism. Brain. 132(Pt 3):604–616. 19153155 10.1093/brain/awn343 PMC2724922

[bhx362C94] Zigmond AS, Snaith RP 1983 The hospital anxiety and depression scale. Acta Psychiatr Scand. 67(6):361–370. 6880820 10.1111/j.1600-0447.1983.tb09716.x

