# Supplementary material for: The Roles of Left Versus Right Anterior Temporal Lobes in Semantic Memory: A Neuropsychological Comparison of Postsurgical Temporal Lobe Epilepsy Patients
Source: Cereb Cortex. 2018 Jan 17;28(4):1487–501. doi: 10.1093/cercor/bhx362 (PMC6093325; doi:10.1093/cercor/bhx362)
Supplement: Supplementary Data [file bhx362_ricecaswellmoorehoffmanlambonralph_supplementarymaterials_cc_revised_final.docx]

## Rice, Caswell, Moore, Hoffman & Lambon Ralph.

## Supplementary Material

**Results**

**Famous Face Naming**

Unlike Drane et al. (2013), we did not find a disproportionate deficit in famous face recognition in right TLE patients. One potential reason for this could be that Drane et al. (2013) excluded any stimuli that patients did not report as familiar to them. To test this possibility, we replicated their “familiar item” analysis in our own data by restricting the analysis to items which participants correctly identified as famous in the familiarity test (see below for full results from this test). Supplementary Figure 2 replots the data including all items as reported in Figure 2, alongside the results from the familiar item analysis. The results for both naming famous people and for famous face recognition remain largely unchanged in the TLE groups and in fact, right TLE patients show even less evidence of differential impairment for famous face recognition.

**Emotion Recognition**

Supplementary Figure 3 shows the results across all expressions and all morphs. Across each of the morph conditions the control participants performed more accurately and faster than the two TLE sub-groups. With the exception of happy expressions (all morphs). To explore the hypothesis that laterality differences may be driven by particular emotions (e.g., a proposed right lateralised dominance for processing fear), the analysis was broken down into the five expressions included in the test for the 100% morphs only (Supplementary Figure 3). In terms of accuracy, a two-way mixed ANOVA with a main effect of Group and Emotion (happy, sad, anger, disgust, fear) showed a significant interaction (F (8, 228) = 2.72, p = 0.007). The interaction was driven by differences in accuracy between the patients and control participants; no differences in accuracy between the left and right TLE groups were found for any expression. Compared to the control group, left TLE patients showed less accurate responses for all expressions except for sadness (Anger: t (38) = 2.90, p = 0.006; Disgust: t (38) = 3.01, p = 0.005; Fear: t (38) = 4.41, p < 0.001; Happy: t (38) = 1.83, p = 0.08). Compared to the control group, right TLE patients showed reduced accuracy for all expressions except for anger (Disgust: t (38) = 3.75, p = 0.001; Fear: t (38) = 2.78, p = 0.008; Happy: t (38) = 1.83, p = 0.08; Sad: t (38) = 2.02, p = 0.05). In terms of reaction times, no significant Group * Expression interaction was found (F (8, 228) = 1.03, p = 0.41).

**Supplementary Figures**

**
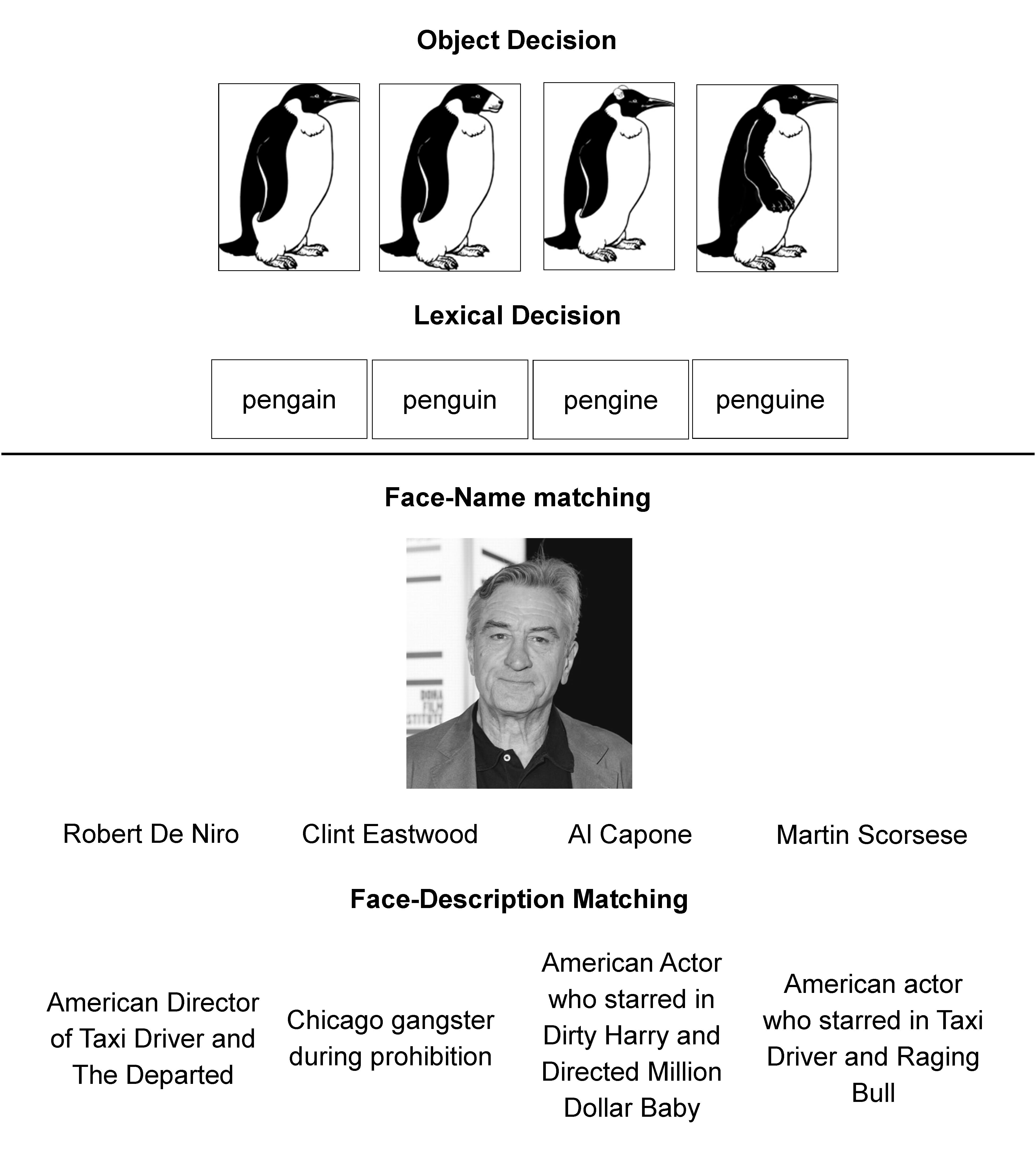
**

**Supplementary Figure 1: Example trials from (A) the object and lexical decision tests and (B) the Face-Name matching test.** For the object and lexical decision tests (top), four stimuli were presented simultaneously and participants had to choose which one was the real object or word. For the face-name matching test (bottom), participants were presented with a picture of a famous face alongside four names. For the face-description matching task, participants had to pick the description that matched the face.


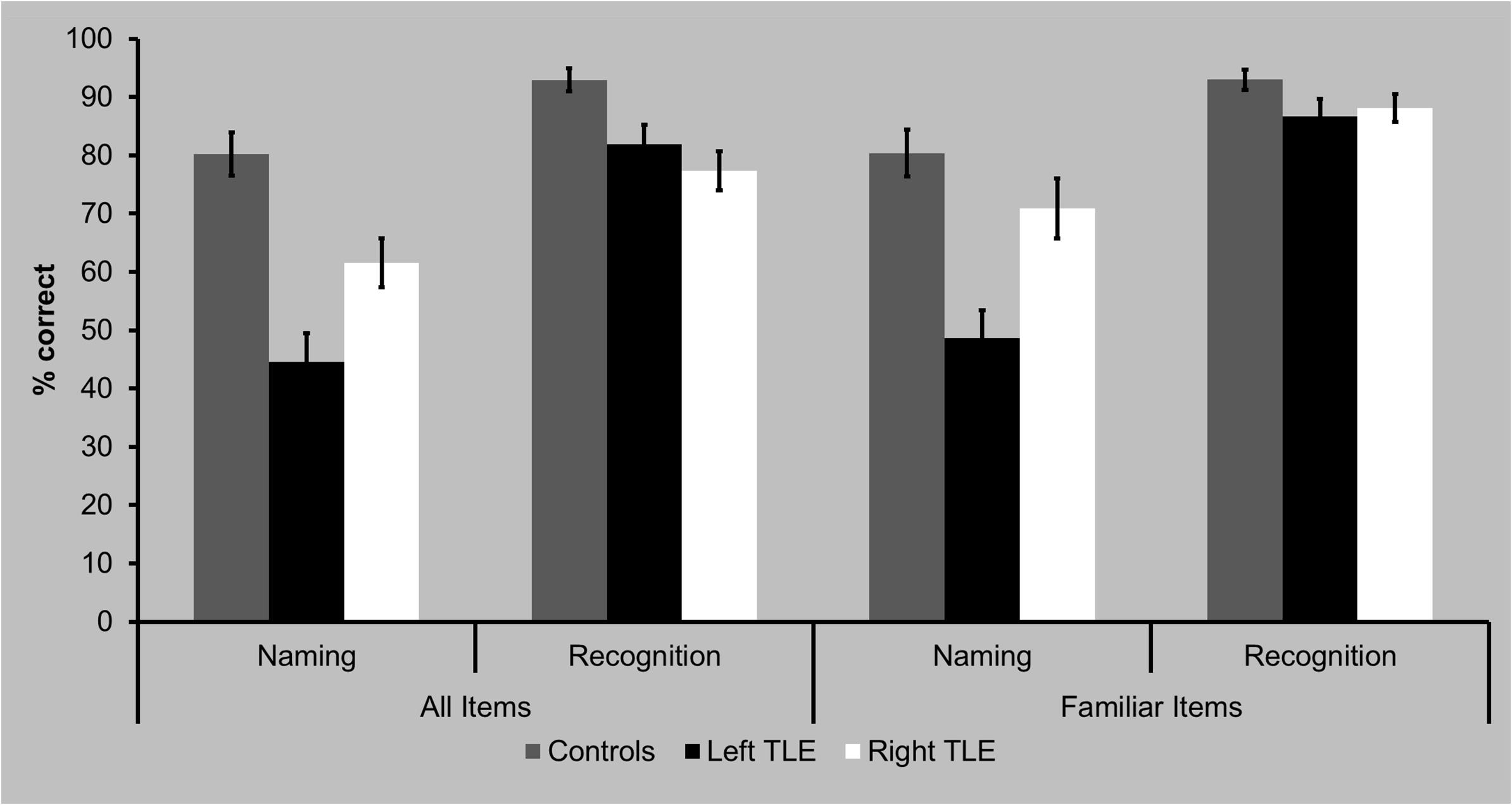


**Supplementary Figure 2: Reanalysis of the Famous Face Naming data.** Comparison of the Famous Face Naming data with all items included versus those that are “familiar” to the participants. The Famous Face Naming and Recognition data from Figure 2a (All Items) are plotted alongside the same data, but restricting analysis to items that participants got correct on the Familiarity test (Familiar Items). Error bars denote standard error.

**
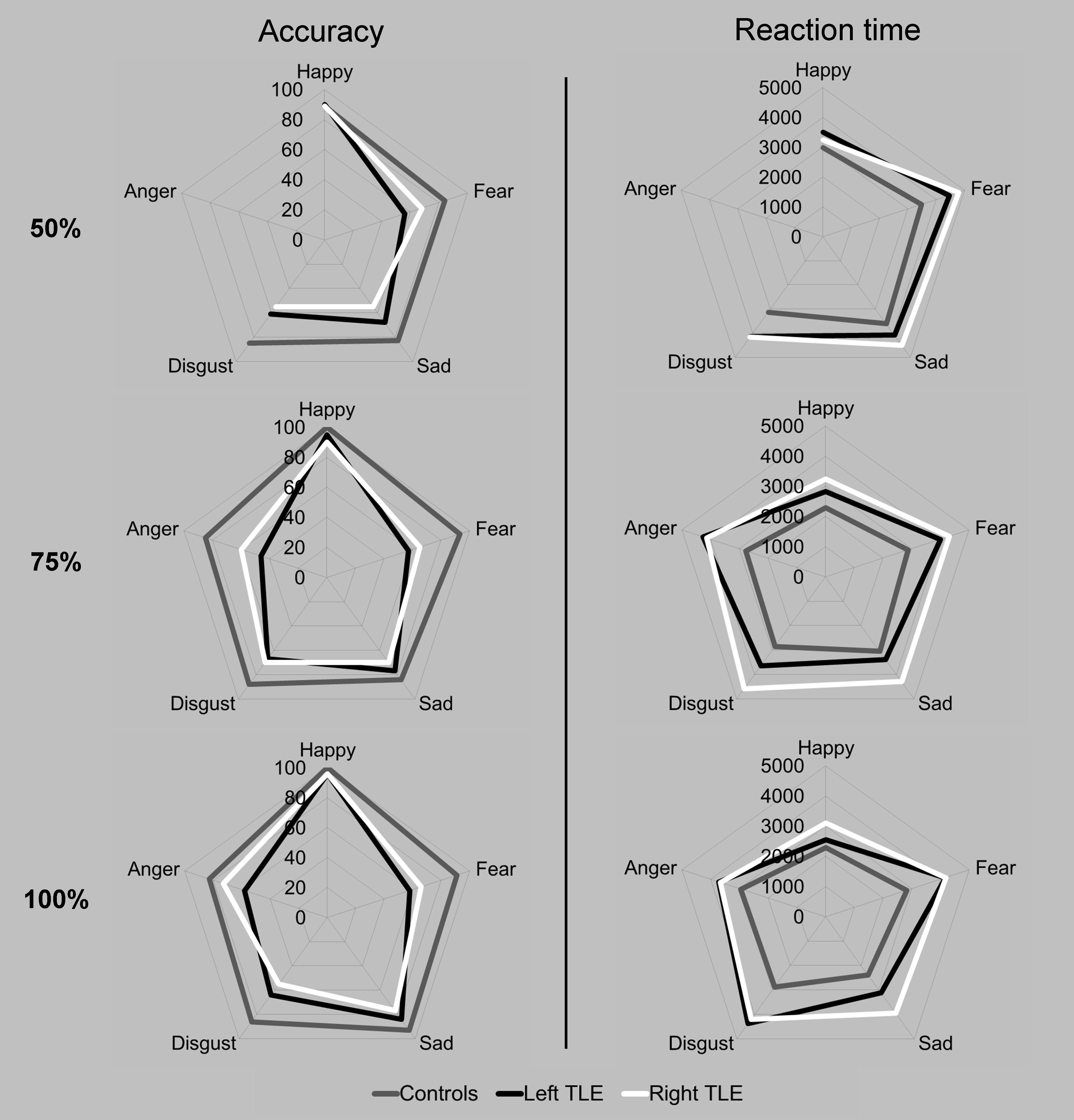
**

**Supplementary Figure 3: Emotion recognition performance broken down by morph**. Accuracy (left) and reaction time (right) data from the 50%, 75% and 100% morphed items are shown for each expression.

| **Supplementary Table 1: Statistical differences between the left and right TLE patients and control participants on each of the semantic and episodic memory tests.** Data shown for accuracy (left) and reaction time (right). The “ANOVA” column reports the main effect of group (left TLE, right TLE, controls). The “planned contrasts” column reports the t-test results between the groups, p-values are Bonferroni corrected for multiple comparisons. Non-significant effects not listed for brevity. The Camel & Cactus test was administered to a subset of patients (left TLE = 16, right TLE = 17) - see main text for details. | | | | |
| --- | --- | --- | --- | --- |
|  | **Accuracy** | | **Reaction Time** | |
|  | **ANOVA** | **Planned Comparisons** | **ANOVA** | **Planned Comparisons** |
| **Camden Faces** | F (2, 57) = 7.69,  p = 0.001 | C > RTLE: t (57) = 3.88, p < 0.001 | F (2, 57) = 15.68,  p < 0.001 | C < LTLE: t (57) = 4.76, p < 0.001  C < RTLE: t (57) = 4.93, p < 0.001 |
| **Camden Words** | F (2, 57) = 5.57,  p = 0.006 | C > LTLE: t (57) = 3.34, p = 0.001 | F (2, 57) = 8.60,  p = 0.001 | C < LTLE: t (57) = 4.05, p < 0.001  C < RTLE: t (57) = 2.79, p = 0.007 |
| **Picture Naming** | F (2, 57) = 12.03,  p < 0.001 | C > LTLE: t (57) = 4.90, p < 0.001  RTLE > LTLE: t (57) = 2.65, p = 0.01 | F (2, 57) = 12.97,  p < 0.001 | C < LTLE: t (57) = 5.09, p < 0.001  C < RTLE: t (57) = 2.59, p = 0.012  RTLE < LTLE: t (57) = 2.50, p = 0.015 |
| **Word-Picture Matching** | F (2, 57) = 8.15,  p = 0.001 | C > LTLE: t (57) = 4.00, p < 0.001 | F (2, 57) = 8.59,  p = 0.001 | C < LTLE: t (57) = 3.92, p < 0.001  C < RTLE: t (57) = 3.12, p = 0.003 |
| **Synonym Judgement** | F (2, 57) = 10.48,  p < 0.001 | LTLE > C: t (57) = 4.20, p < 0.001  RTLE > LTLE: t (57) = 3.69, p = 0.001 | F (2, 57) = 14.03,  p < 0.001 | C < LTLE: t (57) = 4.40, p < 0.001  C < RTLE: t (57) = 4.75, p < 0.001 |
| **Number Judgement** | F (2, 57) = 1.84,  p = 0.17 |  | F (2, 57) = 9.40,  p < 0.001 | C < LTLE: t (57) = 3.14, p = 0.003  C < RTLE: t (57) = 4.16, p < 0.001 |
| **Lexical Decision** | F (2, 57) = 9.95,  p < 0.001 | C > LTLE: t (57) = 4.29, p < 0.001  RTLE > LTLE: t (57) = 3.21, p = 0.002 | F (2, 57) = 15.31,  p < 0.001 | C < LTLE: t (57) = 5.25, p < 0.001  C < RTLE: t (57) = 4.13, p < 0.001 |
| **Object Decision** | F (2, 57) = 5.59,  p = 0.006 | C > RTLE: t (57) = 3.28, p = 0.002 | F (2, 57) = 5.54,  p = 0.006 | C < RTLE: t (57) = 3.22, p = 0.002 |
| **Famous Face Naming** | F (2, 57) = 17.13,  p < 0.001 | C > LTLE: t (57) = 5.85, p < 0.001  C > RTLE: t (57) = 3.05, p = 0.004  RTLE > LTLE: t (57) = 2.81, p = 0.007 | NA | NA |
| **Famous Face Recognition** | F (2, 57) = 7.05,  p = 0.002 | C > LTLE: t (57) = 2.61, p = 0.012  C > RTLE: t (57) = 3.64, p = 0.001 | NA | NA |
| **Face-Name Matching** | F (2, 57) = 7.10,  p = 0.002 | C > LTLE: t (57) = 3.20, p = 0.002  C > RTLE: t (57) = 3.33, p = 0.002 | F (2, 57) = 10.62,  p < 0.001 | C < LTLE: t (57) = 3.27, p = 0.002  C < RTLE: t (57) = 4.49, p < 0.001 |
| **Face-Description Matching** | F (2, 27) = 11.70,  p < 0.001 | C > LTLE: t (57) = 4.44, p < 0.001  C > RTLE: t (57) = 3.89, p < 0.001 | F (2, 57) = 5.50,  p = 0.007 | C < LTLE: t (57) = 2.69, p = 0.009  C < RTLE: t (57) = 3.03, p = 0.004 |
| **Face Familiarity** | F (2, 57) = 12.92,  p < 0.001 | C > RTLE: t (57) = 5.05, p < 0.001  LTLE > RTLE: t (57) = 3.03, p = 0.004 | F (2, 57) = 12.48,  p < 0.001 | C < LTLE: t (57) = 2.82, p = 0.007  C < RTLE: t (57) = 4.98, p < 0.001 |
| **Glasgow Face Matching** | F (2, 57) = 15.28,  p < 0.001 | C > LTLE: t (57) = 4.39, p < 0.001  C > RTLE: t (57) = 5.11, p < 0.001 | F (2, 57) = 9.35,  p < 0.001 | C < RTLE: t (57) = 4.31, p < 0.001 |
| **Emotion Recognition** | F (2, 57) = 14.11,  p < 0.001 | C > LTLE: t (57) = 4.76, p < 0.001  C > RTLE: t (57) = 4.42, p < 0.001 | F (2, 57) = 12.25,  p < 0.001 | C < LTLE: t (57) = 3.56, p = 0.001  C < RTLE: t (57) = 4.76, p < 0.001 |
| **Camel & Cactus (Words)** | F (2, 50) = 3.71, p = 0.03 | C > LTLE: t (50) = 2.53, p = 0.015 | F (2, 49) = 22.17, p < 0.0001 | C < LTLE: T (49) = 5.20, p < 0.0001  C < RTLE: t (49) = 6.04, p < 0.0001 |
| **Camel & Cactus (Pictures)** | F (2, 50) = 0.52, p = 0.60 |  | F (2, 49) = 26.27, p < 0.0001 | C < LTLE: T (49) = 5.57, p < 0.0001  C < RTLE: t (49) = 6.63, p < 0.0001 |

| **Supplementary Table 2: Comparison of statistical differences between the left and right TLE patients before and after controlling for the effect of resection volume.** Data shown for accuracy (left) and correct reaction time (right). The first column re-reports the significance of the full dataset planned comparisons driven by a patient effect (i.e., left TLE > right TLE and vice versa) as reported in Supplementary Table 1. The second column reports the main effect of patient group (left TLE vs. right TLE) in the smaller subset of patients. The third column reports the significance of the main effect of patient group after controlling for resection volume. The cells are colour coded according to the significance of the group main effect. Green indicates significance below p < 0.05, red indicates non-significance, grey indicates data not available. Non-significant results are not reported for brevity. | | | | | | |
| --- | --- | --- | --- | --- | --- | --- |
|  | **Accuracy** | | | **Reaction Time** | | |
|  | **Planned comparison: left TLE, right TLE (TLE=40)** | **ANOVA: left TLE, right TLE (TLE=35)** | **ANCOVA: left TLE, right TLE (TLE=35)** | **Planned comparison: left TLE, right TLE (TLE=40)** | **ANOVA: left TLE, right TLE (TLE=35)** | **ANCOVA: left TLE, right TLE (TLE=35)** |
| **Rey Copy** |  |  |  |  |  |  |
| **Rey Immediate Recall** |  |  |  |  |  |  |
| **Rey Delayed Recall** |  |  |  |  |  |  |
| **Digit Span Forward** |  |  |  |  |  |  |
| **Digit Span Backward** |  |  |  |  |  |  |
| **HADS Anxiety** |  |  | Covariate: F (1, 32) = 4.39, p = 0.04 |  |  |  |
| **HADS Depression** |  |  |  |  |  |  |
| **CBI (overall)** |  |  |  |  |  |  |
| **Camden Faces** |  |  |  |  |  |  |
| **Camden Words** |  |  |  |  |  |  |
| **Picture Naming** | R more accurate than L: t (57) = 2.65, p = 0.01 | Group: F (1, 33) = 5.55, p= 0.03 | Group: F (2, 32) = 5.91, p=0.02  Covariate: F (1, 32) = 0.91, p=0.35 | R quicker than L: t (57) = 2.50, p = 0.015 | Group: F (1, 33) = 3.49, p=0.07 | Group: F (2, 32) = 2.76, p=0.11  Covariate: F (1, 32) = 0.13, p=0.72 |
| **Word-Picture Matching** |  |  |  |  |  |  |
| **Synonym judgement** | R more accurate than L: t (57) = 3.69, p = 0.001 | Group: F (1, 33) = 16.53, p< 0.0001 | Group: F (2, 32) = 5.75, p=0.02  Covariate: F (1, 32) = 1.63, p = 0.21 |  |  |  |
| **Number judgement** |  |  |  |  |  |  |
| **Lexical Decision** | R more accurate than L: t (57) = 3.21, p = 0.002 | Group: F (1, 33) = 3.96, p=0.06 | Group: F (2, 32) = 2.17, p=0.15  Covariate: F (1, 32) = 0.007, p = 0.93 |  |  |  |
| **Object Decision** |  |  |  |  |  | Covariate: F (1, 32) = 4.42, p=0.04 |
| **Famous Face Naming** | R more accurate than L: t (57) = 2.81, p = 0.007 | Group: F (1, 33) = 3.88, p=0.06 | Group: F (2, 32) = 4.14, p=0.05  Covariate: F (1, 32) = 0.66, p= 0.42 |  |  |  |
| **Famous Face Recognition** |  |  | Covariate: F (1, 32) = 5.83, p=0.02 |  |  |  |
| **Face-Name Matching** |  |  | Covariate: F (1,32) = 8.96, p = 0.005 |  |  |  |
| **Face-Description Matching** |  | Group: F (1, 33) = 0.75, p=0.39 | Group: F (2, 32) = 4.45, p=0.04  Covariate: F (1, 32) = 4.96, p=0.03 |  |  |  |
| **Face Familiarity** | L more accurate than R p=0.004 | Group: F (1, 33) = 2.74, p=0.11 | Group: F (2, 32) = 0.61, p=0.44  Covariate: F (1, 32) = 13.37, p=0.001 |  | Group: F (1, 33) = 2.93, p=0.10 | Group: F (2, 32) = 0.01, p=0.91  Covariate: F (1, 32) = 6.04, p=0.02 |
| **Glasgow Face Matching Test** |  |  |  |  |  |  |
| **Emotion Recognition** |  | Group: F (1, 33) = 0.09, p = 0.76 | Group: F (2, 32) = 2.33, p = 0.14  Covariate: F (1, 32) = 4.14, p = 0.05 |  |  |  |
| **Camel & Cactus (Words)** |  |  |  |  |  |  |
| **Camel & Cactus (Pictures)** |  |  |  |  |  |  |
|  | | | | | | |
|  | No RT data | | | | | |
|  | Significant main effect of group (left vs. right TLE) | | | | | |
|  | Main effect of group not significant | | | | | |
